# HSP60 Mediates NLRP3 Inflammasome-Dependent Microglial Pyroptosis Via the TLR4/MyD88/NF-κB Signaling Axis After Subarachnoid Hemorrhage

**DOI:** 10.1007/s10753-025-02442-x

**Published:** 2026-01-12

**Authors:** Zheng-qing Hu, Ruijie Ma, Hang Zhang, Jiahao Miao, Jia-qing Sun, Jinlong Yuan, Jiaqiang Liu, Zihuan Zhang, Dayong Xia

**Affiliations:** 1 Department of Neurosurgery, The Translational Research Institute for Neurological Disorders of Wannan Medical College, the First Affiliated Hospital of Wannan Medical College (Yijishan Hospital of Wannan Medical College), No.2 West Zheshan Road, Wuhu, Anhui 241001 China; 2 Department of Neurosurgery, Affiliated Hospital of Medical School, Nanjing Drum Tower Hospital, Nanjing University, Nanjing, 210002 China

**Keywords:** SAH, Microglia, HSP60, Pyroptosis, NLRP3, Mizoribine

## Abstract

This study aims to elucidate the mechanism by which Heat Shock Protein 60 (HSP60) mediates microglial pyroptosis in the context of early brain injury (EBI) following subarachnoid hemorrhage (SAH), and to investigate the effects of HSP60 inhibition on EBI after SAH. A mouse subarachnoid hemorrhage (SAH) model was established using prechiasmatic cistern blood injection. In vitro, microglia were stimulated with 25 µmol/L oxyhemoglobin (OxyHB) to simulate the SAH pathological environment. In vivo, mice received 100 mg/kg Mizoribine, while in vitro, 80 µmol/L Mizoribine was used to suppress SAH-induced HSP60 upregulation. Techniques including Western blotting, immunofluorescence, immunohistochemistry, transmission electron microscopy, ELISA, modified Garcia neurological scoring, beam walking, brain water content measurement, Morris water maze, TUNEL staining, and Nissl staining were employed to systematically investigate the role of HSP60 inhibition in neuroinflammation and microglial pyroptosis after SAH. Compared to the sham group, both in vivo and in vitro studies with blinded, random sampling of six groups demonstrated a significant increase in HSP60 expression post-SAH. In vivo, 100 mg/kg Mizoribine alleviated blood-brain barrier disruption, cerebral edema, neuronal apoptosis/necrosis, and improved neurological deficits and cognitive impairment. In vitro, 80 µmol/L Mizoribine markedly attenuated microglial activation and pyroptosis, downregulated pro-inflammatory cytokines, and mitigated neuroinflammation. The upregulation of HSP60 after SAH promotes NLRP3 inflammasome assembly by activating the TLR4/MyD88/NF-κB signaling pathway, thereby inducing microglial pyroptosis and exacerbating the progression of early brain injury. Inhibition of HSP60 represents a potential therapeutic strategy for ameliorating EBI after SAH.

**Supplementary Information:**

The online version contains supplementary material available at 10.1007/s10753-025-02442-x.

## Background

This study, using mizoribine, systematically deciphered the spatiotemporal dynamics of HSP60-mediated neuroinflammatory cascades and neuronal injury via the microglial surface TLR4 receptor after subarachnoid hemorrhage (SAH). It further elucidated the molecular network mechanisms during the early brain injury (EBI) phase, ultimately establishing a precise intervention strategy targeting the HSP60/TLR4 signaling axis. This provides a new translational medicine paradigm for improving neurological outcomes in SAH patients.

Subarachnoid hemorrhage (SAH) is an acute cerebrovascular disease with extremely high rates of mortality and disability. The acute-phase mortality remains as high as approximately 35% [1], and about 50% of survivors experience permanent neurological impairments [2], such as cognitive deficits, hemiplegia, and aphasia. Furthermore, the long-term rehabilitation and nursing care required for SAH patients significantly increase the familial and socioeconomic burden [3]. Among the various factors influencing outcomes in SAH patients, early brain injury (EBI) is particularly critical. EBI is generally defined as acute global brain damage occurring within 72 h after SAH onset, and its severity closely correlates with patient mortality, neurological deficits, and the development of later complications ⁠[4]. The pathological mechanisms underlying EBI are highly complex, involving cerebral ischemia, inflammation, oxidative stress, apoptosis, and other processes that interact synergistically, leading to neuronal death and brain tissue damage. Well-established core pathophysiological responses include ischemic injury and energy metabolism dysfunction, blood-brain barrier disruption, neuroinflammation, oxidative stress and ferroptosis, as well as pyroptosis⁠[5–8]. Therefore, in-depth dissection of the molecular network mechanisms of EBI is essential for developing effective intervention strategies targeting the early phase of SAH. However, translational research is still needed to bridge basic research findings to clinical applications in order to overcome current therapeutic limitations.

The dynamic response of microglia following SAH has been identified as a key regulatory node in secondary brain injury. Numerous studies indicate that within minutes after SAH, hemoglobin degradation products (especially heme) in the subarachnoid blood can activate microglia via pattern recognition receptors (e.g., Toll-like receptor 4, TLR4)⁠[9], polarizing them toward a pro-inflammatory (M1) phenotype. Activated M1 microglia release large quantities of pro-inflammatory cytokines such as tumor necrosis factor-α (TNF-α), interleukin-1β (IL-1β), and interleukin-18 (IL-18)⁠[10, 11], driving a significant inflammatory cascade. This excessive inflammatory response not only directly damages neurons but also disrupts blood-brain barrier integrity, promoting vasogenic brain edema and thereby exacerbating EBI [12, 13]. Pyroptosis is a recently identified novel form of programmed, pro-inflammatory cell death mediated by inflammasome activation and Gasdermin proteins (e.g., GSDMD). Unlike apoptosis, pyroptosis is characterized by plasma membrane pore formation and rupture, leading to the massive release of inflammatory contents and triggering intense local and systemic immune responses. Under pathological conditions in the central nervous system, activated microglia are a major cell type susceptible to pyroptosis [14, 15].Microglial membrane Toll-like receptors (TLR4) recognize Pathogen-Associated Molecular Patterns (PAMPs) or Damage-Associated Molecular Patterns (DAMPs), activating Nuclear Factor kappa B (NF-κB) signaling via the Myeloid Differentiation Factor 88 (MyD88)-dependent pathway. This initiates transcription of pro-inflammatory genes (including NLRP3, pro-IL-1β, and pro-IL-18) [16, 17]. Subsequently, NLRP3, ASC, and pro-caspase-1 assemble into the NLRP3 inflammasome complex, which catalyzes pro-caspase-1 auto-cleavage into enzymatically active caspase-1. Activated caspase-1 has two critical functions: cleaving pro-inflammatory cytokine precursors (pro-IL-1β and pro-IL-18) into mature, bioactive IL-1β and IL-18, enabling their robust secretion; and specifically cleaving Gasdermin D (GSDMD) to generate its N-terminal domain (GSDMD-N). GSDMD-N translocates to the plasma membrane and oligomerizes into transmembrane pores [18, 19]. Pore formation increases membrane permeability, causing ion influx (Na⁺, Cl⁻, H₂O), osmotic imbalance, cell swelling, and lytic cell death (pyroptosis). This process facilitates massive release of intracellular inflammatory cytokines (IL-1β, IL-18), amplifying local and systemic inflammation [20].

In subarachnoid hemorrhage (SAH), microglial phenotypic transformation is a central driver of neuroinflammation. Mechanistically, microglial TLR4 activates the NF-κB pathway, critically regulating neuroinflammation initiation and maintenance [21]. Recent studies identify Heat Shock Protein 60 (HSP60), released by damaged tissues as an endogenous DAMP, as a specific ligand for microglial TLR4, triggering NF-κB-dependent inflammatory cascades [22–24]. Beyond its conserved chaperone role in protein folding, HSP60 exerts pleiotropic roles in stress response, inflammation activation, cell death regulation, and disease progression. While constitutively expressed under physiological conditions, HSP60 is significantly upregulated in pathological microenvironments (precancerous lesions, neurodegenerative diseases) [25]. However, HSP60-mediated microglial activation mechanisms in SAH remain elusive and require further investigation. Given HSP60’s essential role in cellular homeostasis, genetic knockout is impractical. Thus, the specific inhibitor mizoribine was employed to target its pathological overexpression phase [26–28].

## Methods and Materials

### Animals and SAH Models

Male C57BL/6J mice (weight: 25 ± 2 g; age: 6–7 weeks) were provided by the Experimental Animal Center of Yijishan Hospital, Wannan Medical College. All experimental procedures were approved by the Animal Ethics Committee of Yijishan Hospital, Wannan Medical College (Approval No.: WNMC-AWE-2024224) and strictly followed the National Institutes of Health Guide for the Care and Use of Laboratory Animals. The mice were housed under barrier conditions at a temperature of 25 ± 1 °C and relative humidity of 55 ± 5%, with a 12-hour light/dark cycle, and provided with adequate food and water ad libitum.

The SAH model was induced using the prechiasmatic cistern injection method [29]. Mice were anesthetized via intraperitoneal injection of pentobarbital (40 mg/kg), with the depth of anesthesia confirmed by the loss of corneal reflex. The mouse was placed in a prone position on a stereotaxic instrument. A 1 cm sagittal scalp incision was made, and the subcutaneous fascia was bluntly dissected. A 1 mm diameter craniotomy was drilled 4.5 mm posterior to the bregma along the midline to expose the dura mater. Arterial blood (50 µL) was collected via cardiac puncture from a donor mouse under anesthesia. For injection into the prechiasmatic cistern, a microinjection needle (Hamilton, #81000, USA) was inserted vertically through the craniotomy to a depth of 3.5 mm, and 50 µL of arterial blood was slowly infused (0.5 µL/min). The needle was left in place for 2 min and then withdrawn gradually to prevent reflux. The craniotomy site was sealed with bone wax, and the skin was sutured layer by layer and disinfected with iodine. Sham-operated control mice underwent identical procedures including dural penetration but without blood injection. After surgery, animals were placed on a warming blanket maintained at 37 °C for 45 min during recovery before being returned to standard housing cages. Neurological deficits were assessed using the modified Garcia scoring system, and mice with a score < 6 were excluded from the study.

## Cellular and in Vitro Model Culture

Primary microglia were isolated from the brains of neonatal mice within 24 h after birth. The meninges were carefully removed under a microscope to retain cortical tissues. The brain tissues were finely minced and placed in a petri dish on ice, then further dissected into small pieces and transferred into trypsin digestion solution (Solarbio, #T1300, China). Tissues were digested for 5 min at 37 °C in an incubator. The digestion was terminated by adding high-glucose DMEM medium (Gibco, #6125123,America) supplemented with 10% fetal bovine serum (Opcel, #BS-1102, China). The digested tissues were transferred to a new dish and thoroughly minced. The homogenate was filtered through a 40 μm strainer, centrifuged at 1000 × g for 5 min, and resuspended before seeding into culture flasks for primary culture. The cells were maintained in high-glucose DMEM containing 10% FBS and 1% penicillin-streptomycin solution (Solarbio, #P1400, China). The medium was completely replaced on day 3 and half replaced on day 7. After 10 days of adherent culture, suspended microglial cells were collected using a thermostatic shaking incubator (150 rpm, 37 °C, 1 h), centrifuged, and seeded into experimental plates. In this study, primary microglia were stimulated with 25 µmol/L oxyhemoglobin (OxyHb; Solarbio, #H8020, China)⁠[30] to establish an in vitro SAH model.

## Drug Administration

Mizoribine (MCE, #HY-17470, China) was dissolved in physiological saline at graded concentrations. Mice were anesthetized with intraperitoneal pentobarbital (40 mg/kg) and fixed in a stereotaxic apparatus. A 10 µL Hamilton syringe (Hamilton, #80900, USA) was inserted into the lateral ventricle through a cranial burr hole at the following coordinates: 1.5 mm lateral and 1.0 mm posterior to the bregma, at a depth of 3.5 mm. an injection volume of 1 µL. An infusion pump delivered the drug at a constant rate of 0.5 µL/min. The needle was left in place for 10 min after infusion to prevent backflow and then slowly withdrawn over 5 min. The burr hole was sealed thoroughly with bone wax. The HSP60 inhibitor myrtucommulone A (Aladdin, #54247-21−1, China) was administered via intraperitoneal injection at a dose of 4.5 mg/kg. The compound’s activity as an HSP60 inhibitor has been previously described [49], and the dosing regimen was selected based on established protocols in anti-inflammatory studies [50].

Intrathecal injection of 40 µg HSP60 (MCE, # HY-P73916A, China) can specifically activate the TLR4-MyD88 pathway, and was used to establish a model of neuroinflammatory brain injury [51].

### Experimental Program in Figure 1

**Fig. 1:**
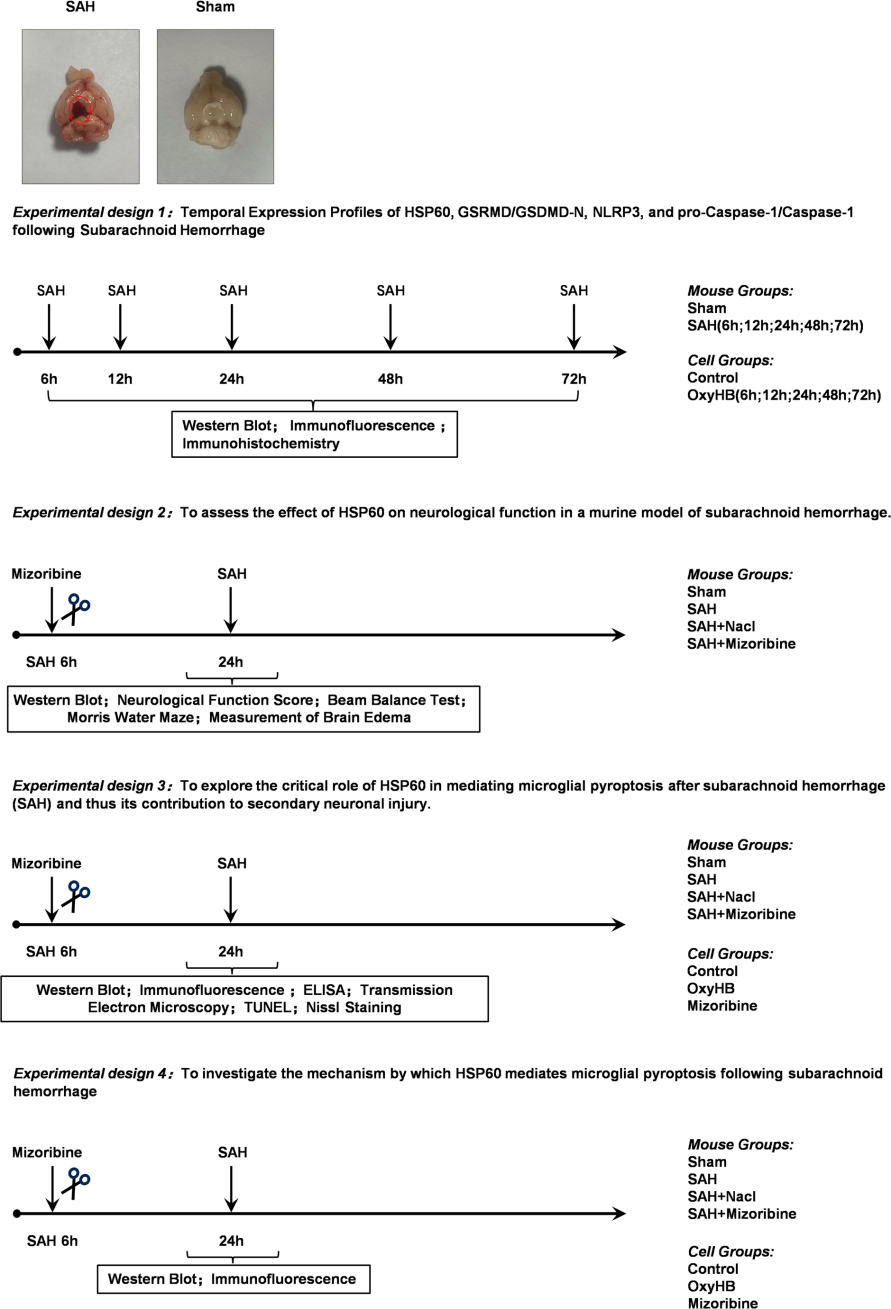
Experimental Design and Grouping: Both in vivo and in vitro subarachnoid hemorrhage (SAH) models were established to investigate the functional role of HSP60. The in vivo experiment utilized a mouse SAH model divided into the following groups: Sham, SAH, SAH + NaCl, and SAH + Mizoribine. The in vitro experiment employed primary microglia grouped as Control, OxyHB, and OxyHB + Mizoribine, to systematically evaluate the regulatory mechanism of HSP60 in neuroinflammation following SAH

All animals were randomly assigned to four independent experimental protocols, with randomization carried out at the time of enrollment into each study. For immunofluorescence staining and imaging experiments, investigators remained blinded to the selection of slides for microscopic evaluation. In animal experiments, strict randomization and blinding protocols were implemented throughout. For group allocation, an independent researcher assigned mice to the Sham, SAH, SAH + Vehicle, and SAH + Mizoribine groups using computer-generated random numbers following model induction; this researcher was only responsible for recording assignments and did not participate in subsequent procedures. The order of all interventions—including drug administration, behavioral testing, and tissue collection—was also randomized. To ensure blinding during interventions, Mizoribine and an equal volume of vehicle were prepared and coded by an independent individual, so that the personnel performing the injections remained unaware of group identities. For outcome assessment, all evaluations involving subjective analysis—such as neurological scoring, behavioral tests, and histological examinations (e.g., TUNEL staining, Nissl staining, and immunohistochemistry)—were conducted independently by at least two researchers blinded to group allocation, with all samples de-identified prior to assessment.

### Experimental Design 1

To evaluate the temporal expression changes of HSP60, GSDMD/GSDMD-N, NLRP3, and pro-Caspase-1/Caspase-1 following SAH, animals were randomly divided into six groups (Sham, SAH 6 h, SAH 12 h, SAH 24 h, SAH 48 h, and SAH 72 h; *n* = 10 per group). The SAH model was induced, and mice were euthanized at designated time points (6 h, 12 h, 24 h, 48 h, and 72 h post-SAH). Six mice from each group were selected for Western blot analysis, and the remaining mice were used for immunofluorescence staining. For the in vitro arm of the study, microglial cells were stimulated with oxyhemoglobin (OxyHB), and the grouping strategy mirrored that used in the animal experiments.

### Experimental Design 2

To determine the optimal dose of Mizoribine, mice were randomly assigned to seven groups (Sham, SAH, SAH + Vehicle, SAH + Mizoribine 50 mg/kg, SAH + Mizoribine 100 mg/kg, SAH + Mizoribine 150 mg/kg, and SAH + Mizoribine 200 mg/kg; *n* = 10 per group). Mizoribine was administered intracerebroventricularly 6 h after SAH induction. Mice were euthanized at pre-defined time points. Six mice per group were used for Western blot and immunohistochemical analysis to evaluate HSP60 inhibition and identify the optimal dose of Mizoribine.

For in vitro studies, the CCK-8 assay was first employed to determine the concentration range of Mizoribine that enhanced cell viability. Subsequently, effective concentrations of Mizoribine (20 µM, 40 µM, and 80 µM) were applied to identify the optimal dose for inhibiting HSP60 release in microglial cells.

To evaluate the effect of HSP60 on neurological function in SAH mice, the animals were randomly divided into four groups (Sham, SAH, SAH + Vehicle, and SAH + Mizoribine; *n* = 50 per group). Mizoribine was administered via intracerebroventricular injection 6 h after SAH. Neurological function scoring, beam walking test, Morris water maze test, and brain water content measurement were performed at 24 h post-SAH. Six mice from each group were randomly selected for Western blot analysis of tight junction proteins (ZO-1 and Occludin).

### Experimental Design 3

To investigate the key role of HSP60 in microglial pyroptosis after SAH and the impact of pyroptosis on neurons, relevant markers were examined, including ASC, NLRP3, GSDMD/GSDMD-N, pro-Caspase-1/Caspase-1, pro-IL-1β/IL-1β, and CD86/CD206. Mice were randomly divided into four groups (Sham, SAH, SAH + Vehicle, and SAH + Mizoribine; *n* = 50 per group). Western blot, immunofluorescence staining, ELISA, and transmission electron microscopy were used to detect the relevant markers. TUNEL staining and Nissl staining were applied to assess the correlation between microglial pyroptosis and neuronal injury. For each detection method, six mice were randomly selected from each group. For in vitro studies, microglial cells were divided into three groups (Control, OxyHB, and OxyHB + Mizoribine), and pyroptosis-related markers were similarly evaluated.

### Experimental Design 4

To explore the potential mechanism by which HSP60 mediates microglial pyroptosis after SAH, mice were randomly assigned to four groups (Sham, SAH, SAH + Vehicle, and SAH + Mizoribine; *n* = 20 per group). Western blot and immunofluorescence staining were performed to detect relevant signaling markers, including TLR4, MyD88, P65, and p-P65. For each assay, six mice were randomly selected from each group. The in vitro grouping was consistent with that in Experimental Design 3, and Western blot and immunofluorescence staining were similarly used to detect molecular signaling markers.

### Brain Edema Measurement

To evaluate the extent of cerebral edema, the wet-dry weight method was employed to measure brain water content. Specifically, mice from each group were euthanized immediately after SAH induction, and whole brains were rapidly harvested. The wet weight of each intact brain was recorded promptly. Brain samples were then placed in a constant-temperature oven at 60 °C for 24 h. After complete drying, samples were cooled to room temperature and their dry weights were accurately measured. The brain water content was calculated using the following formula: [(wet weight − dry weight)/wet weight] × 100%.

### Behavioral Assessment

All behavioral tests were performed by an investigator blinded to the experimental groups.

### Modified Garcia Score for Neurological Deficits

Neurological sensorimotor deficits were quantitatively assessed at 24 and 72 h after SAH induction using an 18-point modified Garcia scoring system. This scoring system integrates six independent behavioral domains: spontaneous activity, axial stability, forelimb extension, climbing reflex, bilateral trunk tactile response, and bilateral vibrissae-elicited response. Each domain is scored on a 0–3 point scale (3: intact function; 0: complete absence of function) based on the degree of functional preservation. The total score, ranging from 3 to 18, is the sum of the scores from all six domains, with a lower score indicating more severe neurological impairment.

### Beam Walking Test for Motor Coordination and Balance

Motor coordination and balance were quantitatively evaluated using a standardized beam apparatus (1 m in length, 10 mm in diameter). Each mouse underwent three independent trials, and the average score from these trials was used as the final result. The scoring criteria were as follows: 0 points: inability to grip the beam or immediate fall; 1 point: ability to grip the beam but no movement within 60 s; 2 points: ability to move a short distance (< 10 cm) on the beam but falling within 60 s; 3 points: ability to traverse the entire length of the beam but with incidents of slipping or falling; 4 points: free and steady traversal of the beam without any slipping or falling events [31].

### Morris Water Maze

Spatial learning and reference memory were assessed using the Morris water maze, beginning one week after SAH surgery. The test was conducted over five consecutive days. During the first four days (hidden platform training), mice were placed into the water from four different starting points each day, and the time required to locate the submerged hidden platform within a 60-second limit (escape latency) was recorded. If a mouse failed to find the platform within the time limit, it was gently guided to the platform and allowed to remain there for 10 s. On the fifth day, a probe trial was conducted: the platform was removed, and the mouse was placed in the water at the midpoint of the quadrant opposite the original platform location and allowed to swim freely for 60 s. The entire process was recorded using a video tracking system (EthoVision XT, Noldus, Netherlands), and parameters including escape latency, time spent in the target quadrant, and swimming path were quantified and analyzed.

### Immunofluorescence Staining (IF)

After model establishment, mice were transcardially perfused with ice-cold PBS buffer (KGL, #KGL2206-500, China) followed by 4% paraformaldehyde fixative (Biomiky, #MK014A, China). The whole brain was carefully dissected and post-fixed in 4% paraformaldehyde at 4 °C for 24 h. Tissues were then dehydrated in sequential 15% and 30% sucrose solutions until they settled at the bottom. After removing surface moisture, 12 μm thick coronal frozen sections were prepared using a cryostat. Sections were air-dried at 37 °C for 30 min, and tissue areas were circled with a hydrophobic barrier pen. After washing with PBS (3 × 5 min), sections were permeabilized with 0.3% Triton X-100 (Beyotime, #P0096, China) for 30 min at room temperature and blocked with immunofluorescence blocking buffer (Beyotime, #P0260, China) for 1 h. Primary antibodies were applied and incubated overnight at 4 °C in a humidified chamber. After PBS washes (3 × 5 min), sections were incubated with species-appropriate fluorescent secondary antibodies for 2 h at room temperature, followed by additional PBS washes. Finally, sections were mounted using an anti-fade mounting medium containing DAPI (Beyotime, #P0131, China). For in vitro studies, microglial cells grown on poly-L-lysine-coated coverslips or glass-bottom dishes were fixed with 4% PFA, permeabilized, blocked, and incubated with primary antibodies (4 °C, overnight) and corresponding fluorescent secondary antibodies (room temperature, 2 h). After PBS washes, cells were mounted with DAPI. Fluorescence imaging was performed using a Pannoramic MIDI fluorescence microscope.

### Nissl Staining

Using the same sectioning procedure as for IF, Nissl staining was performed strictly according to the instructions of the Cresyl Violet Staining Kit (Solarbio, #G1430, China). Sections were immersed in Cresyl Violet solution for 15 min, rinsed with distilled water to remove excess dye, differentiated in Nissl differentiation solution for 2 min, dehydrated rapidly in absolute ethanol, cleared in xylene, and mounted with neutral balsam. Imaging was conducted using a Pannoramic MIDI slide scanner.

### Immunohistochemistry (IHC)

Tissue samples were fixed in 4% paraformaldehyde. Endogenous peroxidase activity was quenched with 3% hydrogen peroxide. Non-specific binding sites were blocked with 5% BSA for 30 min at room temperature. Sections were incubated with specific primary antibodies at 4 °C overnight in a humidified chamber. After PBS washes (3 × 5 min), sections were incubated with biotin-labeled secondary antibodies for 30 min at room temperature. Following another round of PBS washes, streptavidin-horseradish peroxidase complex was applied for 20 min at room temperature. DAB substrate was used for color development (1–3 min), with the process monitored under a microscope. Nuclei were counterstained with hematoxylin for 1 min. Sections were dehydrated through graded ethanol, cleared in xylene, and mounted with neutral balsam. Stained sections were scanned using a Pannoramic MIDI slide scanner.

### Cell Viability Analysis

To evaluate the dose-dependent effect of mizoribine on microglial cells and determine its optimal effective concentration, cell viability was quantitatively assessed using the Cell Counting Kit-8 (CCK-8) (NCM Biotech, #C6005, China). Cells treated with various concentrations of mizoribine in 96-well plates were incubated for 24 h. CCK-8 solution was then added, and incubation continued for 2 h. The optical density (OD) at 450 nm was measured using a microplate reader. Cell viability was expressed as a percentage relative to the untreated control group.

### TUNEL Assay

Following the immunofluorescence staining procedure (including fixation, permeabilization, blocking, and primary antibody incubation), neuronal apoptosis was detected using a one-step TUNEL Apoptosis Detection Kit (Beyotime, #C1089, China) according to the manufacturer’s instructions. NeuN-immunolabeled frozen sections were incubated in the dark with TUNEL reaction mixture containing fluorescein-dUTP for 60 min at room temperature. After thorough PBS washes, sections were mounted with anti-fade mounting medium containing DAPI. Imaging was performed using a Pannoramic MIDI fluorescence microscope with excitation/emission wavelengths of approximately 550/570 nm (for TUNEL signal) and the DAPI channel to assess apoptotic events in NeuN-positive neurons.

### Western Blotting

#### Co-Immunoprecipitation (Co-IP)

To investigate the interaction between HSP60 and TLR4, co-immunoprecipitation was performed. Microglial cells were lysed on ice for 10 min using the Co-IP Kit (#P2179S, Beyotime, China) containing protease and phosphatase inhibitors. The lysates were centrifuged at 14,000 g for 15 min at 4 °C, and the supernatant was collected. Twenty microliters (20 µL) of the lysate were reserved as the Input control. The remaining supernatant was incubated with 10 µL of Protein G magnetic beads and 3 µL of either anti-HSP60 or anti-TLR4 antibody. The mixture was incubated on a rotary shaker overnight at 4 °C. The immunoprecipitated complexes were then subjected to Western blot analysis.

### Nuclear and Cytoplasmic Fractionation

Nuclear and cytoplasmic proteins were isolated using the Nuclear and Cytoplasmic Protein Extraction Kit (#P0028, Beyotime, China). According to the manufacturer’s protocol, cytoplasmic extraction reagents A and B were sequentially added to the collected cell pellets. The cytoplasmic fraction was extracted by vigorous vortexing followed by centrifugation at 14,000 g for 15 min at 4 °C, and the supernatant was transferred to a fresh microcentrifuge tube. The remaining pellet was then treated with nuclear extraction reagent to isolate nuclear proteins, repeating the same steps as described for cytoplasmic extraction.

Brain tissue and cellular samples were lysed on ice using RIPA lysis buffer (Beyotime, #P0013B, China) containing protease and phosphatase inhibitor cocktails (Beyotime, #P1045, China) for 30 min. Lysates were centrifuged at 14,000 rpm for 15 min, and the supernatant was collected as total protein extract. Protein concentration was determined using a BCA Protein Assay Kit (Beyotime, #P0010, China), and samples were normalized to equal concentrations. Protein samples (typically 20–50 µg) were mixed with loading buffer and denatured by boiling at 100 °C for 10 min. Denatured proteins were separated by SDS-PAGE and transferred onto PVDF membranes (Merck Millipore, #IPVH00010, USA) using a wet transfer system at constant current. After transfer, membranes were blocked with 5% non-fat milk in Tris-buffered saline containing 0.1% Tween-20 (TBST) for 2 h at room temperature. Membranes were washed with TBST (3 × 10 min) and incubated with appropriately diluted primary antibodies at 4 °C overnight. The following day, membranes were washed again with TBST (3 × 10 min) and incubated with species-specific horseradish peroxidase (HRP)-conjugated secondary antibodies for 2 h at room temperature. After final TBST washes (3 × 10 min), membranes were incubated with enhanced chemiluminescence (ECL) substrate (Merck Millipore, #WKLS0500, USA). Chemiluminescence signals were captured and analyzed using a Western blot imaging system. Details regarding the primary and secondary antibodies used are provided in Table 1.

**Table 1:** Antibodies used in the article

Antibody	Product Code	Application	Dilution Ratio	Company	Area
HSP60	12165	WB IF IHC	1: 2000 1: 200 1: 200	CST	America
ZO-1	21773-1-AP	WB	1: 2000	proteintech	China
Occludin	91131	WB	1: 2000	CST	America
TLR4	P60712R1S	WB IF	1: 2000 1: 200	abmart	China
MyD88	4283T	WB IF	1: 2000 1: 200	CST	America
Phospho-NF-κB p65	82335-1-RR	WB IF	1: 2000 1: 200	proteintech	China
NF-κB p65	T55034S	WB	1: 2000	abmart	China
NLRP3	P60622R3S	WB IF	1: 2000 1: 200	abmart	China
Caspase-1	P79884R2	WB IF	1: 2000 1: 200	abmart	China
Gasdermin D	PU224937S	WB IF	1: 2000 1: 200	abmart	China
ASC	67824T	WB IF	1: 2000 1: 200	CST	America
IL-1β	P50520-1R1S	WB	1: 2000	abmart	China
NeuN	94403	IF	1: 200	CST	America
GAPDH	81640-5-RR	WB	1: 10000	proteintech	China
Beta Actin	81115-1-RR	WB	1: 10000	proteintech	China
Histone H3	17168-1-AP	WB	1: 10000	proteintech	China
Beta Tubulin	2146	WB	1: 10000	CST	America
CD86	TD6332S	IF	1: 200	abmart	China
CD206	81525-1-RR	IF	1: 200	Proteintech	China
Iba1	ab289874	IF	1: 200	abcam	America
HRP-Goat Anti-Mouse	RGAM001	WB	1: 5000	Proteintech	China
HRP-Goat Anti-Rabbit	RGAR001	WB IHC	1: 5000 1: 200	Proteintech	China
Alexa Fluor™ 488	A32766	IF	1:400	Invitrogen	America
Alexa Fluor™ 647	A-21447	IF	1:400	Invitrogen	America
Alexa Fluor™ 594	A-21207	IF	1:400	Invitrogen	America

### Enzyme-Linked Immunosorbent Assay (ELISA)

The levels of IL-1β and IL-18 in mouse brain homogenates and microglial cell suspensions after subarachnoid hemorrhage were detected using commercial mouse IL-1β (Yuanju Bio, #YJ301814, China) and IL-18 (Realgen Bio, #RGM180-2, China) ELISA kits. All experimental procedures were strictly performed in accordance with the manufacturers’ instructions.

### Transmission Electron Microscopy (TEM)

Pyroptosis is characterized by ultrastructural features including cell swelling, chromatin margination and condensation, plasma membrane blebbing, cytoplasmic vacuolization, and endoplasmic reticulum dilation, ultimately leading to plasma membrane pore formation and rupture [32]. To assess microglial pyroptosis, transmission electron microscopy was employed to observe these morphological alterations. Brain tissue samples were immediately immersed in 2.5% glutaraldehyde solution (Biosharp, #BL911A, China) for primary fixation (room temperature, 2–4 h). After fixation, tissues were thoroughly rinsed with PBS (3 × 10 min) and then post-fixed in 1% osmium tetroxide (OsO₄) solution (room temperature, 2 h), followed by additional PBS rinses (3 × 10 min). Tissue blocks were dehydrated through a graded series of ethanol (50%, 70%, 80%, 90%, 95%, 100%) and acetone. Dehydrated samples were infiltrated for approximately 12 h in a mixture of acetone and epoxy resin embedding medium (SPI, #90529–77 − 4, China), then embedded and polymerized at 60 °C for 48 h. Ultrathin Sect. (70 nm thick) were cut using an ultramicrotome. Sections were double-stained with uranyl acetate and lead citrate and observed under a transmission electron microscope (FEI Tecnai G2, USA) to identify characteristic ultrastructural features of pyroptosis in microglial cells.

### Statistical Analysis

Data analysis and visualization were performed using GraphPad Prism 9 software (GraphPad Software, USA). All quantitative data are expressed as mean ± standard deviation. Comparisons among multiple groups were conducted using one-way analysis of variance (ANOVA), and intergroup differences were analyzed with Tukey’s post hoc test. A value of *P* < 0.05 was considered statistically significant.

## Results

1. The expression of HSP60 and pyroptosis markers both reach their peak at 24 h after SAH

**Fig. 2:**
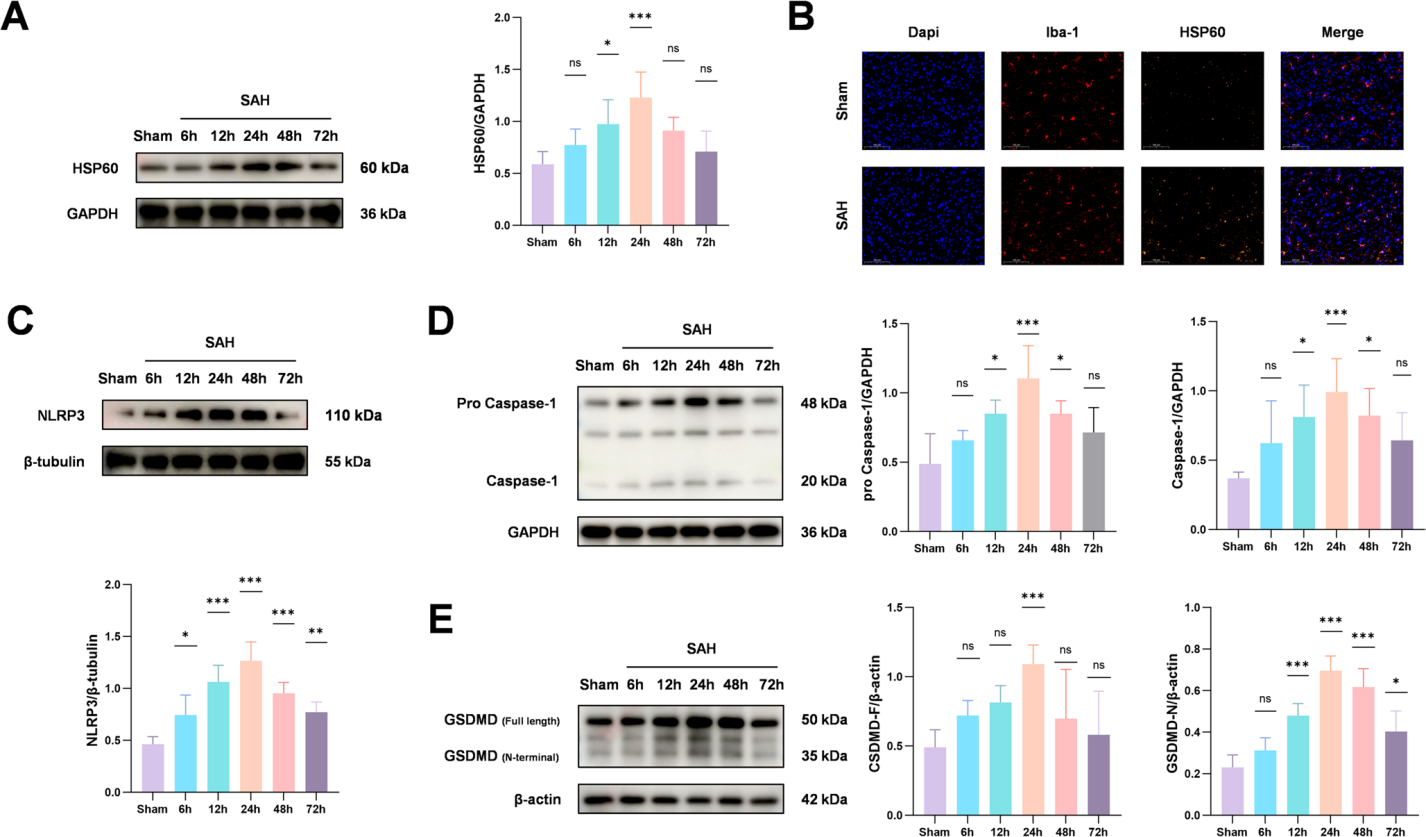
To investigate the molecular mechanisms underlying early brain injury (EBI) after SAH, we established an in vivo SAH model in mice. The protein expression dynamics of HSP60, NLRP3, Caspase-1, and GSDMD in the ipsilateral cortex were analyzed at various time points using Western blot analysis. The results showed that compared with the sham-operated group, the expression levels of these proteins were significantly increased at 24 h after SAH (Fig. 2A, C, D, E). Further immunofluorescence co-localization analysis revealed a marked increase in the number of HSP60-positive cells in the brain tissue of the SAH group compared to the Sham group (Fig. 2B). One-way analysis of variance (ANOVA) followed by Tukey’s post hoc test was performed for comparisons among multiple groups. Data are presented as mean ± SD; ns indicates not significant, *P < 0.05, **P < 0.01, ***P < 0.001

**Fig. 3:**
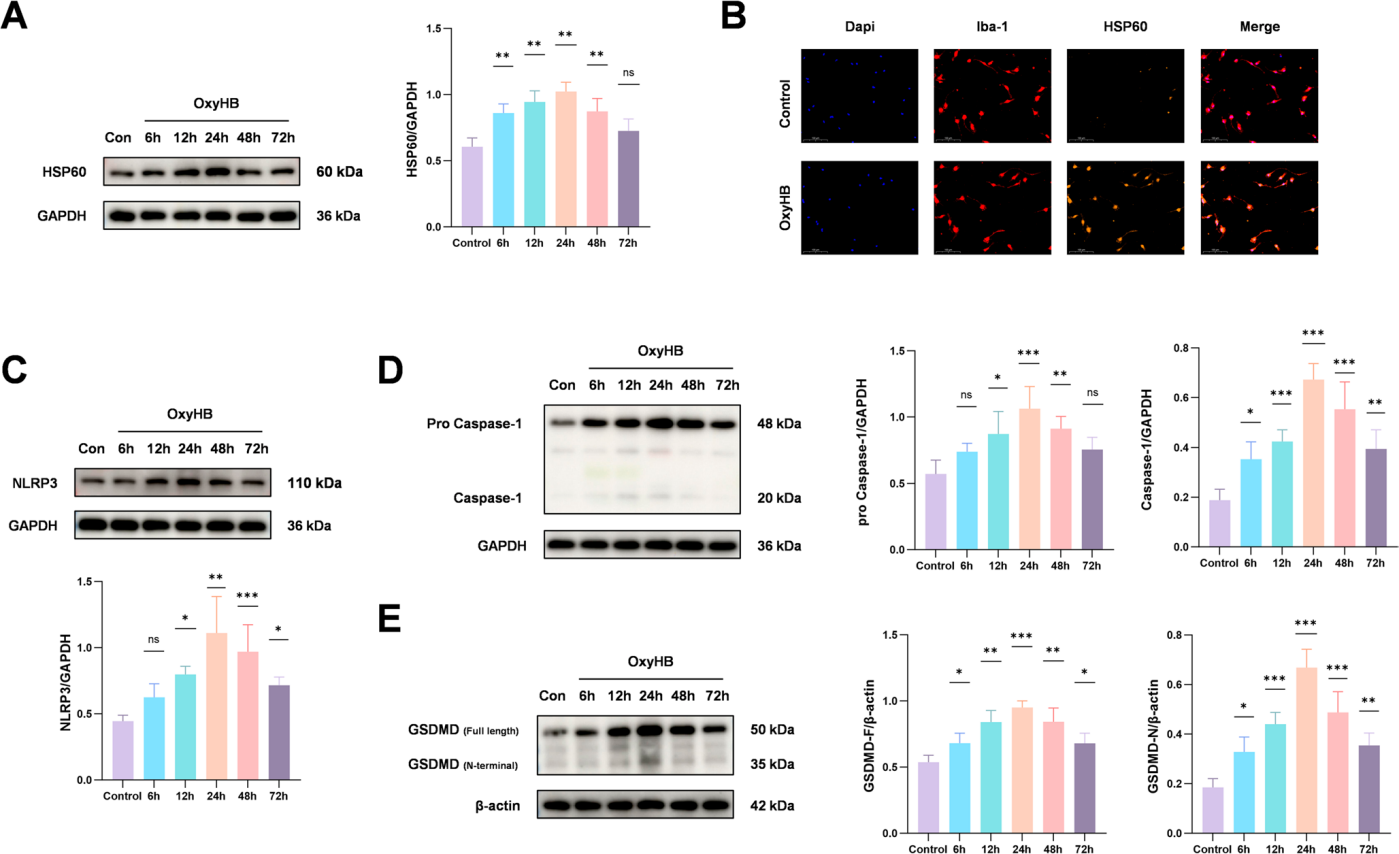
To simulate the pathological process following SAH at the cellular level and investigate its underlying molecular mechanisms, we established an in vitro SAH model by stimulating primary microglia with oxyhemoglobin (OxyHB). Western blot analysis revealed that, compared to the blank control group, the protein expression levels of HSP60, NLRP3, Caspase-1, and GSDMD were significantly upregulated after 24 h of OxyHB stimulation (Fig. 3A, C, D, E). Consistent with this, immunofluorescence co-localization results showed a pronounced increase in the number of HSP60-positive cells in the OxyHB-stimulated group compared to the blank control group (Fig. 3B). One-way analysis of variance (ANOVA) followed by Tukey’s post hoc test was performed for comparisons among multiple groups. Data are presented as mean ± SD; ns indicates not significant, *P < 0.05, **P < 0.01, ***P < 0.001

To investigate the pathological role of HSP60 in early brain injury after subarachnoid hemorrhage (SAH), we first detected the expression level of HSP60 in the ipsilateral cortical brain tissue of mice from each group. Western blot analysis revealed that the protein level of HSP60 began to increase at 6 h post-SAH, rose significantly by 24 h, and subsequently declined gradually (Fig. 2A). Colocalization analysis of microglia and HSP60 via immunofluorescence staining showed a marked increase in HSP60 expression within the microglia of SAH model mice compared to the sham-operated group (Fig. 2B). In in vitro experiments, we treated primary microglia with oxyhemoglobin (OxyHB) to simulate SAH conditions. Western blot results demonstrated a significant upregulation of HSP60 expression after 24 h of OxyHB stimulation (Fig. 3A). Immunofluorescence staining further confirmed a substantial enhancement of HSP60 fluorescence intensity in OxyHB-treated primary microglia (Fig. 3B).To elucidate the role of microglial pyroptosis in early brain injury after SAH, we examined the expression of key pyroptosis-related molecular markers (NLRP3, Caspase-1, and GSDMD) in the ipsilateral cortex. Western blot results indicated that the levels of these pyroptosis-related proteins were all significantly elevated at 24 h after SAH induction (Fig. 2C、D、E). Consistent with the in vivo findings, primary microglia treated with OxyHB for 24 h also exhibited a significant increase in the protein expression of NLRP3, Caspase-1, and GSDMD (Fig. 3C、D、E).

These results indicate that during SAH-induced early brain injury, the expression level of HSP60 demonstrated a clear temporal correlation with the occurrence of microglial pyroptosis, with both HSP60 and pyroptosis markers reaching their peak expression at 24 h post-SAH.

2. Mizoribine effectively suppresses SAH-induced upregulation of HSP60 during early brain injury

**Fig. 4:**
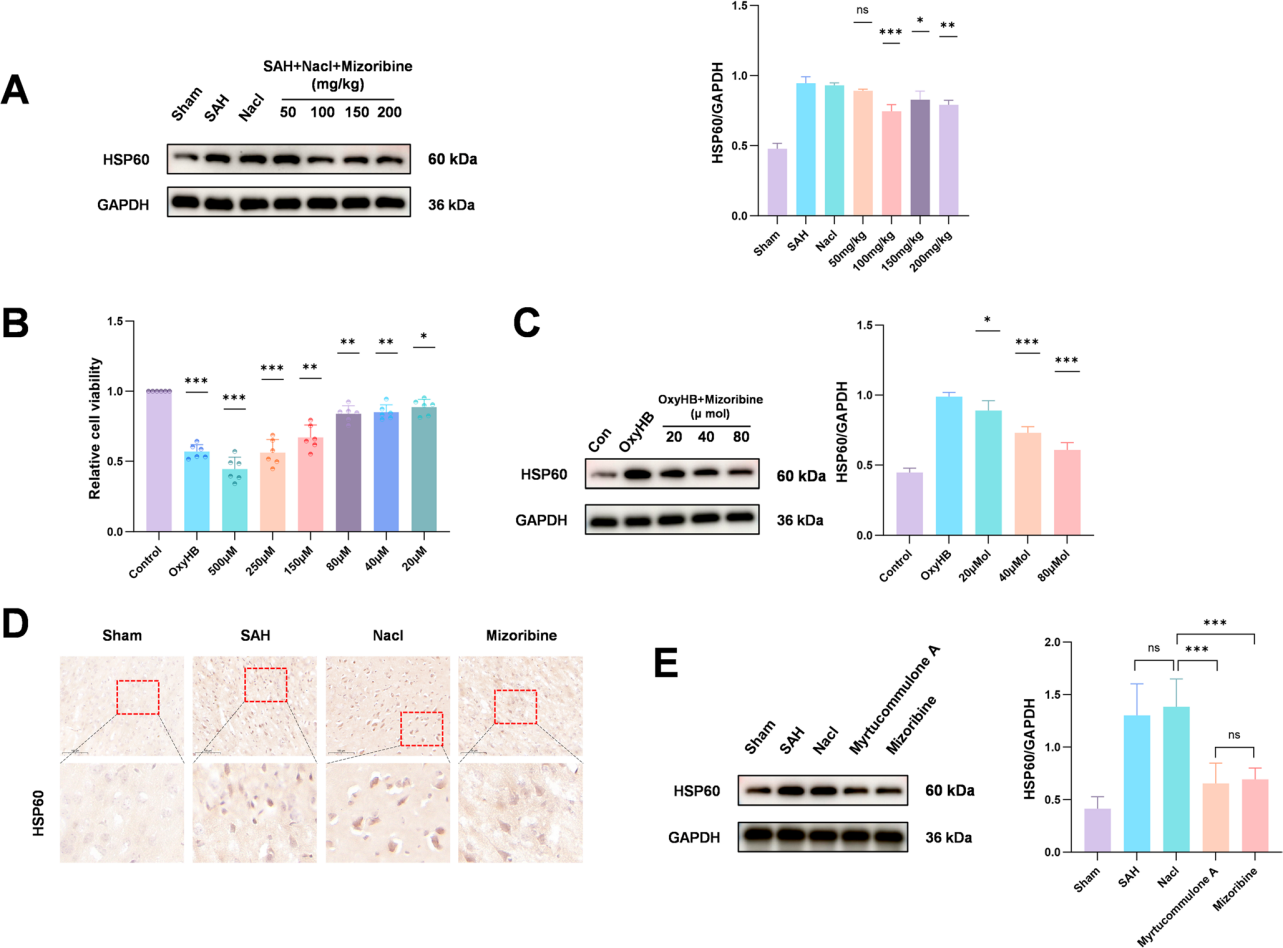
To determine the optimal concentration of Mizoribine for inhibiting HSP60, Western blot was performed to assess the effect of different concentrations of Mizoribine on HSP60 expression. The results showed that the most significant inhibition of HSP60 was achieved at a dose of 100 mg/kg (Fig. 4A). Additionally, the CCK-8 assay revealed that Mizoribine concentration-dependently restored microglial viability under OxyHB stimulation (Fig. 4B). Further validation by Western blot confirmed that 80 µmol/L was the optimal concentration under in vitro conditions (Fig. 4C). Immunohistochemical results further indicated that Mizoribine treatment significantly reduced the abnormally elevated HSP60 expression in the SAH model (Fig. 4D). Furthermore, the efficacy of Mizoribine was further validated using another HSP60 inhibitor, Myrtucommulone A(Fig. 4E). One-way analysis of variance (ANOVA) followed by Tukey’s post hoc test was performed for comparisons among multiple groups. Data are presented as mean ± SD; ns indicates not significant, *P < 0.05, **P < 0.01, ***P < 0.001

Mizoribine, an immunosuppressive agent, potently inhibits HSP60 expression. To determine its optimal dose for suppressing HSP60 in the SAH-induced EBI model, we evaluated a concentration gradient in vivo. Western blot results demonstrated that 100 mg/kg Mizoribine most effectively inhibited HSP60 expression (Fig. 4A). Furthermore, the inhibitory effect of Mizoribine on HSP60 was further validated using another HSP60 inhibitor, Myrtucommulone A (Fig. 4E). Immunohistochemical staining further confirmed that Mizoribine treatment significantly reduced HSP60-positive expression compared to the SAH + NaCl group (Fig. 4D). In vitro, we first assessed cell viability using the CCK-8 assay to identify the optimal concentration of Mizoribine. Results showed that Mizoribine at 20 µM, 40 µM, and 80 µM significantly attenuated the OxyHB-induced reduction in microglial viability (Fig. 4B). Subsequent Western blot analysis revealed that 80 µM Mizoribine most effectively inhibited HSP60 expression (Fig. 4C).

3. Inhibition of HSP60 attenuates NLRP3 inflammasome-induced microglial pyroptosis after SAH

**Fig. 5:**
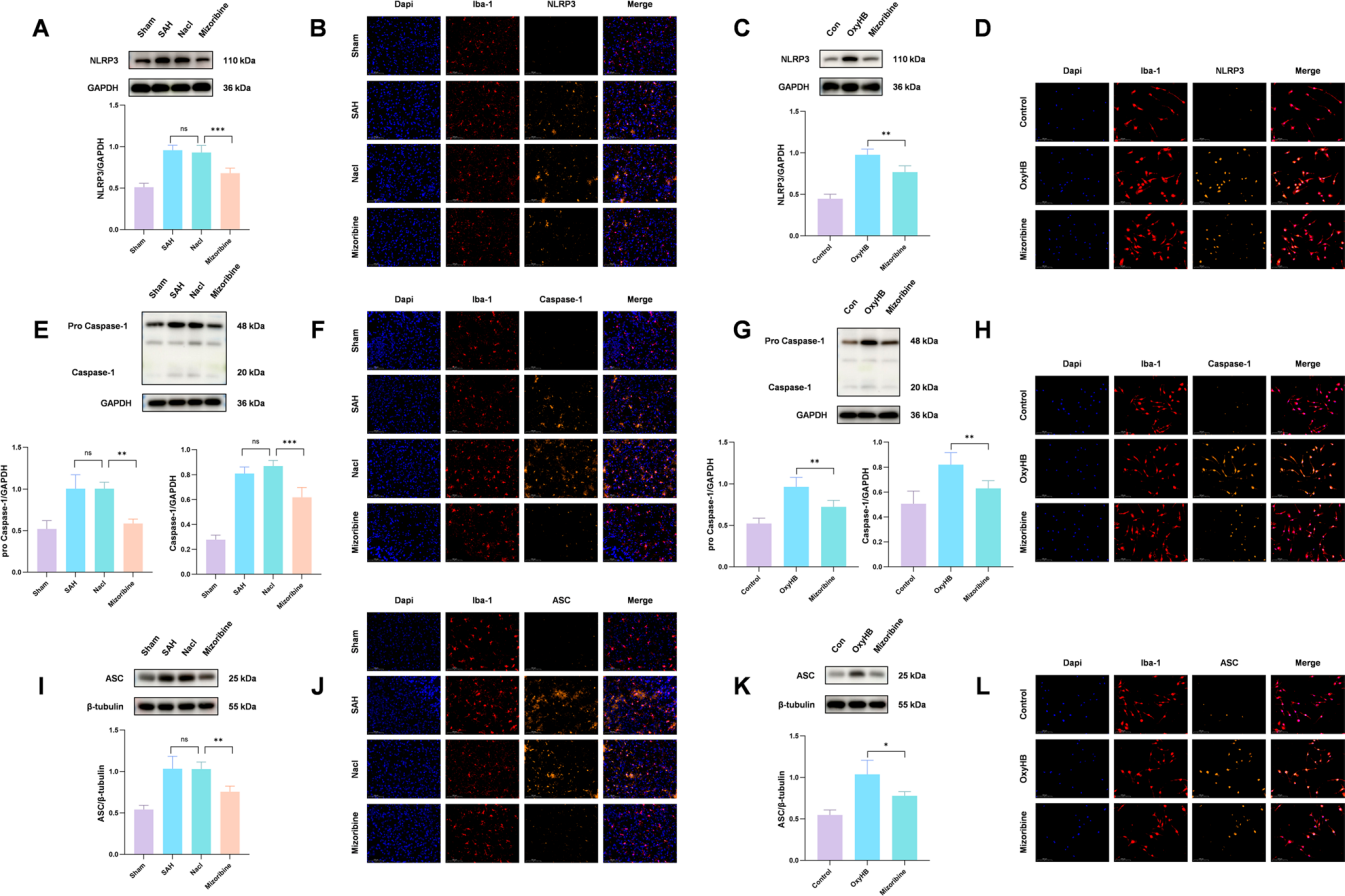
To investigate the role of HSP60 in microglial pyroptosis after SAH, the expression of key proteins in the NLRP3 inflammasome pathway, including NLRP3, pro-Caspase-1/Cleaved Caspase-1, and ASC, was detected by Western blot. In vivo results showed that inhibition of HSP60 with Mizoribine significantly reduced the expression levels of these proteins (Fig. 5A, E, I). A consistent trend was observed in cellular experiments (Fig. 5C, G, K). For further verification, immunofluorescence double staining was performed to label microglia and related target proteins in both in vivo and in vitro models. The changes in protein expression were consistent with the Western blot results (Fig. 5B, F, J, D, H, L). In summary, Mizoribine treatment significantly suppressed the activation of the NLRP3 inflammasome after SAH, indicating that HSP60 is involved in regulating SAH-induced microglial pyroptosis. One-way analysis of variance (ANOVA) followed by Tukey’s post hoc test was performed for comparisons among multiple groups. Data are presented as mean ± SD; ns indicates not significant, *P < 0.05, **P < 0.01, ***P < 0.001

The inflammasome multiprotein complex, composed of NLRP3, ASC, and pro-caspase-1, plays a critical role in pyroptosis. Western blot analysis showed that Mizoribine-mediated inhibition of HSP60 significantly reduced the expression of NLRP3, Caspase-1, and ASC proteins in the SAH + Mizoribine group compared to the SAH + NaCl group (Fig. 5A, E, I). Immunofluorescence co-staining of microglia with these target proteins confirmed a marked reduction in positive cells after Mizoribine treatment (Fig. 5B, F, J). Consistent results were obtained in vitro: Western blot analysis showed that Mizoribine treatment effectively suppressed OxyHB-induced upregulation of NLRP3, Caspase-1, and ASC (Fig. 5C, G, K), which was supported by immunofluorescence in primary microglia (Fig. 5D, H, L).

**Fig. 6:**
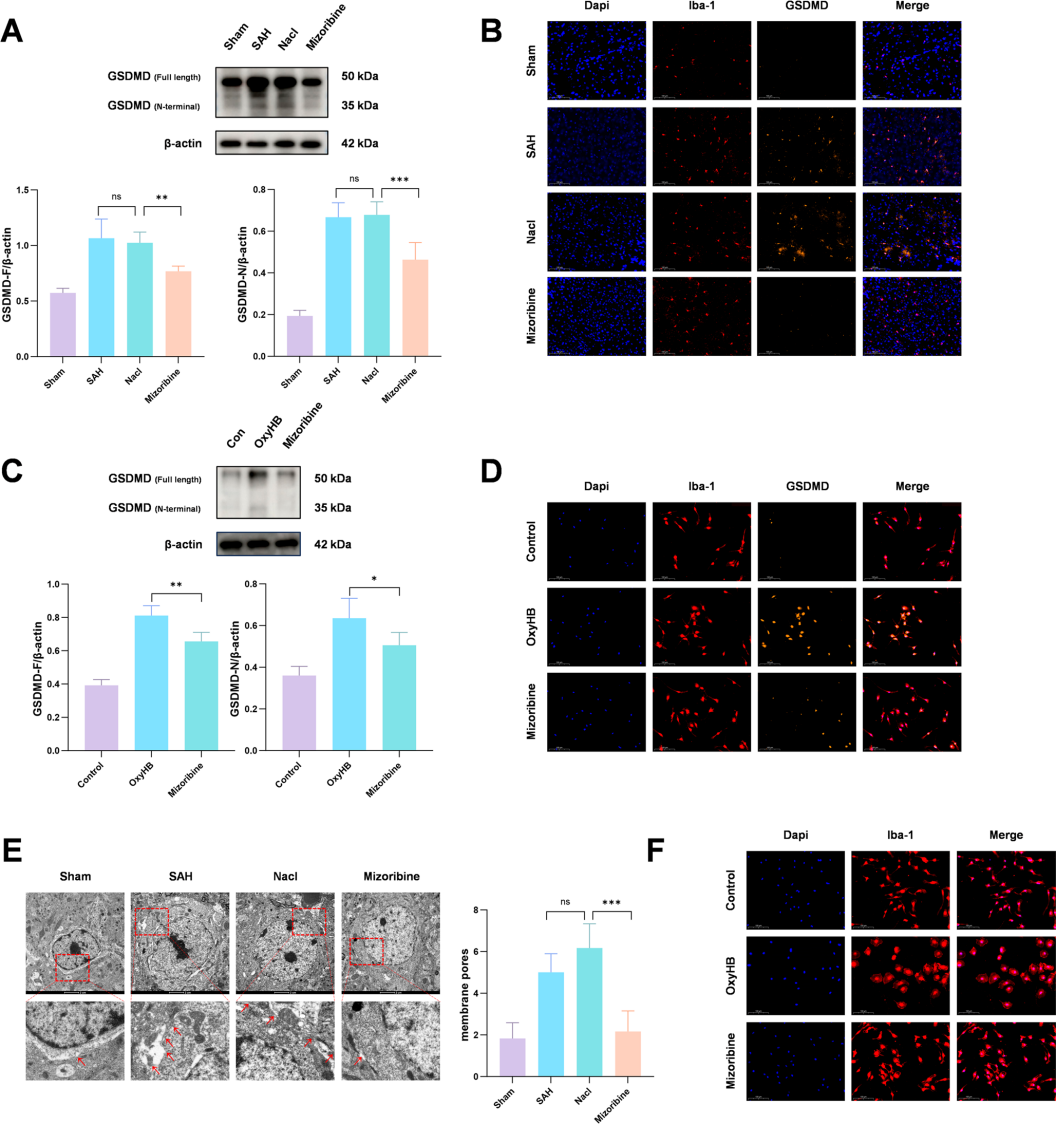
To further explore the mechanism of HSP60 in microglial pyroptosis, the expression of GSDMD-N, the key cleaved and activated form of GSDMD that mediates plasma membrane pore formation, was detected. Western blot analysis showed that the protein levels of GSDMD-F/GSDMD-N were significantly reduced in the Mizoribine treatment group compared with the SAH group, a finding consistently validated in both in vivo and in vitro experiments (Fig. 6A, C). Further co-localization analysis of microglia via immunofluorescence revealed a marked reduction in positive signals for the target proteins in the Mizoribine group (Fig. 6B, D). Transmission electron microscopy observations indicated that Mizoribine treatment significantly ameliorated ultrastructural alterations associated with pyroptosis, including plasma membrane pore formation (red arrows) (Fig. 6E). Additionally, assessment of plasma membrane integrity in primary microglia by immunofluorescence staining demonstrated that inhibition of HSP60 markedly alleviated SAH-induced membrane integrity damage (Fig. 6F). One-way analysis of variance (ANOVA) followed by Tukey’s post hoc test was performed for comparisons among multiple groups. Data are presented as mean ± SD; ns indicates not significant, *P < 0.05, **P < 0.01, ***P < 0.001

To further investigate the relationship between HSP60 and microglial pyroptosis, we assessed the expression of GSDMD and its active form GSDMD-N, which mediates membrane pore formation. Western blot showed that Mizoribine significantly reduced both GSDMD and GSDMD-N expression compared to the SAH + NaCl group (Fig. 6A). Immunofluorescence also indicated fewer GSDMD-positive microglia in the Mizoribine group (Fig. 6B). In vitro, Mizoribine treatment suppressed GSDMD and GSDMD-N expression relative to the OxyHB group (Fig. 6C), consistent with immunofluorescence findings (Fig. 6D). Moreover, transmission electron microscopy (TEM) analysis of microglial ultrastructure revealed that, compared to the Sham group, cells in both the SAH and SAH + NaCl groups exhibited disrupted plasma membrane integrity and the formation of membrane pores. These pathological alterations were significantly ameliorated by Mizoribine intervention. (Fig. 6E). In an in vitro model using primary microglia cultured in serum-free medium, immunofluorescence observations also showed consistent results: the OxyHB group displayed plasma membrane rupture, whereas Mizoribine treatment effectively alleviated membrane damage (Fig. 6F). These results demonstrate that inhibition of HSP60 effectively attenuates microglial pyroptosis.

4. Inhibition of HSP60 alleviates neuroinflammation and reduces activation of pro-inflammatory microglia after SAH

**Fig. 7:**
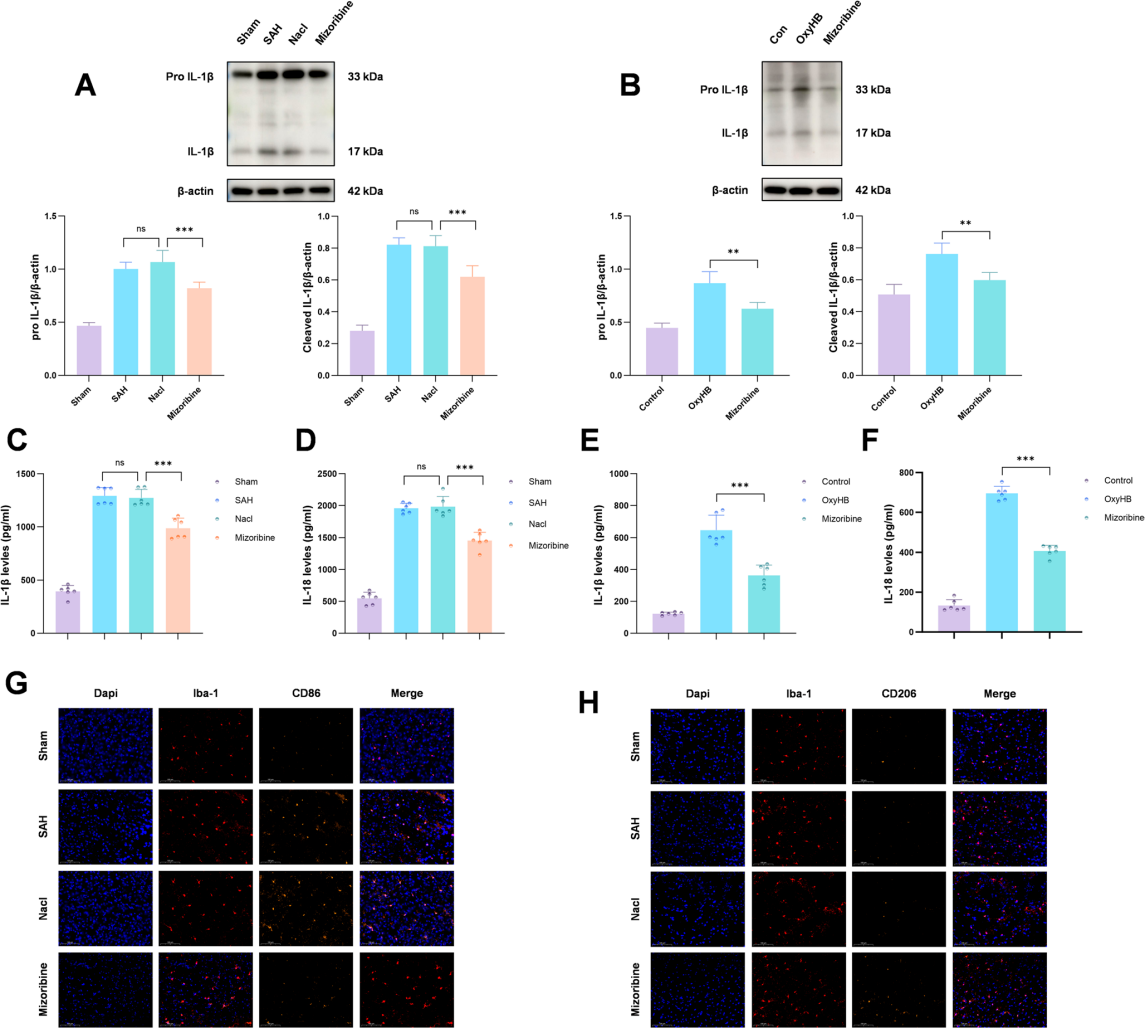
The expression levels of inflammatory cytokines following microglial pyroptosis were detected by Western blot. The results showed that the expression of IL-1β and its cleaved form, Cleaved IL-1β, was significantly increased after SAH, while Mizoribine treatment effectively inhibited the expression of both proteins (Fig. 7A, B). Further evaluation of the concentrations of IL-18 and IL-1β by ELISA revealed that Mizoribine significantly reduced the SAH-induced elevation of both cytokines (Fig. 7C–F). Moreover, immunofluorescence detection of microglial activation showed that inhibition of HSP60 reduced the number of M1-positive microglia and promoted an increase in M2-type microglia (Fig. 7G, H). One-way analysis of variance (ANOVA) followed by Tukey’s post hoc test was performed for comparisons among multiple groups. Data are presented as mean ± SD; ns indicates not significant, **P < 0.01, ***P < 0.001

Pyroptosis leads to the release of inflammatory factors. Western blot analysis showed that IL-1β levels were significantly elevated after SAH but reduced following Mizoribine treatment (Fig. 7A). A similar trend was observed in vitro, where Mizoribine inhibited OxyHB-induced IL-1β upregulation (Fig. 7B). ELISA confirmed that Mizoribine reduced IL-1β and IL-18 levels both in vivo (Fig. 7C, D) and in vitro (Fig. 7E, F).

Microglia polarize into pro-inflammatory M1 and anti-inflammatory M2 phenotypes [33]. Immunofluorescence co-staining showed that SAH increased CD86-positive (M1) microglia, which was reversed by Mizoribine (Fig. 7G). Conversely, CD206-positive (M2) microglia increased after Mizoribine treatment (Fig. 7H). These results indicate that inhibiting HSP60 mitigates neuroinflammation, reduces M1 microglial activation, and promotes M2 polarization.

5. ***Inhibition of HSP60 ameliorates neuronal damage***,*** improves neurobehavioral outcomes***,*** and reduces blood-brain barrier disruption after SAH***

**Fig. 8:**
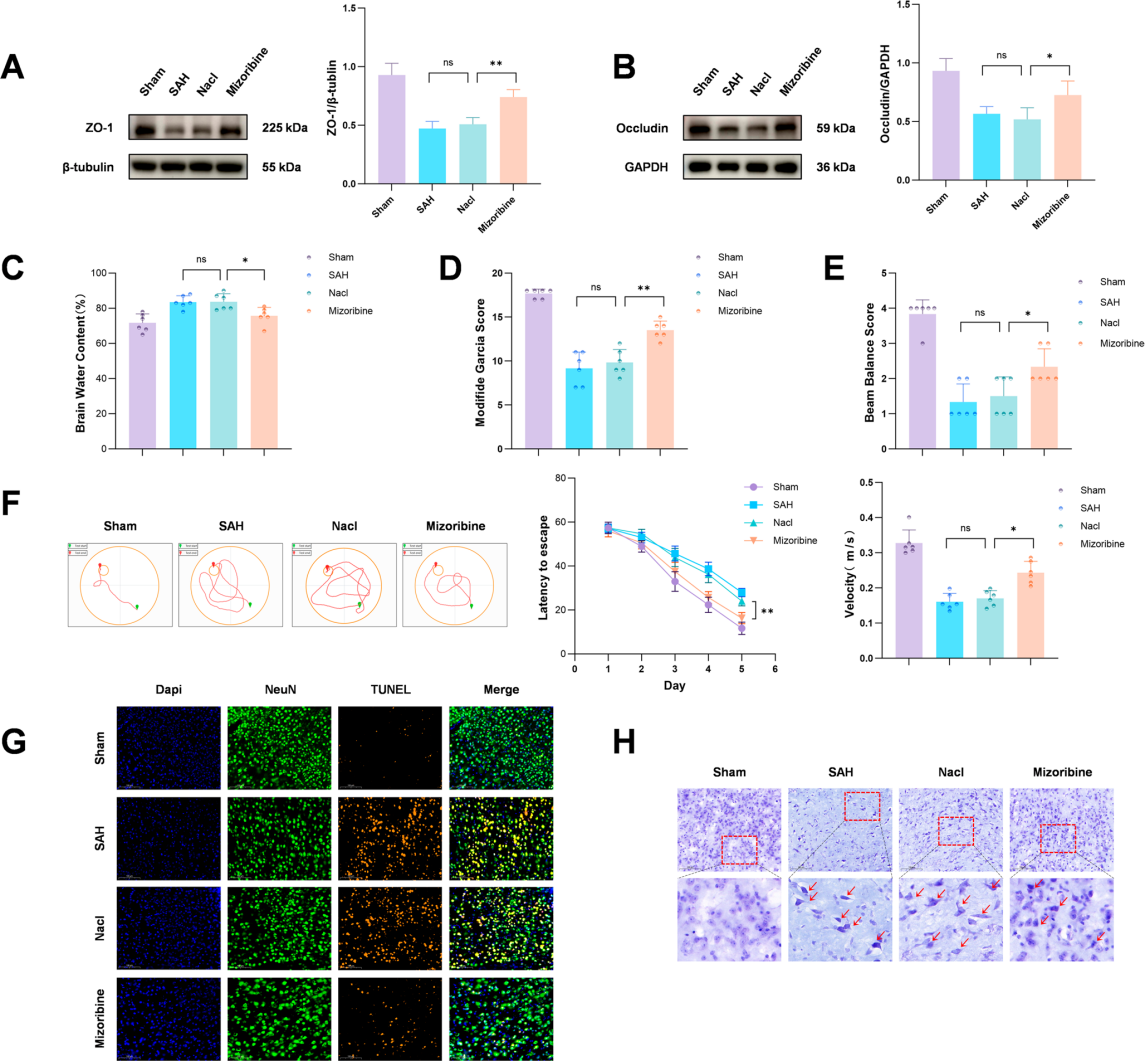
Western blot analysis of the expression levels of blood-brain barrier tight junction proteins ZO-1 and Occludin showed a significant decrease after SAH, while Mizoribine intervention markedly alleviated the SAH-induced downregulation of these proteins (Fig. 8A, B). Measurement of brain water content indicated that Mizoribine treatment effectively reduced cerebral edema after SAH (Fig. 8C). Neurological function assessments, including the modified Garcia score, beam walking test, and water maze test, demonstrated that mice treated with the HSP60 inhibitor Mizoribine achieved higher scores in all behavioral tests, indicating significant improvement in neurological deficits (Fig. 8D–F). Further evaluation of neuronal damage by TUNEL staining and Nissl staining revealed that inhibition of HSP60 significantly reduced neuronal apoptosis and improved Nissl body integrity after SAH, suggesting a neuroprotective effect of Mizoribine treatment (Fig. 8G, H). One-way analysis of variance (ANOVA) followed by Tukey’s post hoc test was performed for comparisons among multiple groups. In addition, two-way repeated measures ANOVA were applied to analyze the data of long-term neurological functions. Data are presented as mean ± SD; ns indicates not significant, *P < 0.05, **P < 0.01

Western blot analysis showed that SAH reduced the expression of tight junction proteins (occludin and ZO-1), which was reversed by Mizoribine (Fig. 8A, B). Brain water content was significantly increased in the SAH group but attenuated by Mizoribine (Fig. 8C). Neurobehavioral assessments, including the modified Garcia score, beam walking test, and Morris water maze, revealed significant deficits in the SAH + NaCl group compared to sham controls. Mizoribine treatment improved Garcia scores (Fig. 8D), beam walking performance (Fig. 8E), and spatial learning and memory in the water maze, with shorter escape latencies and path lengths (Fig. 8F). Through TUNEL staining and Nissl staining to assess neuronal injury, it was found that extensive neuronal damage existed in the cortical area of the SAH + NaCl group, while Mizoribine intervention effectively reduced the level of this damage. (Fig. 8G). These results indicate that HSP60 contributes to neuronal damage post-SAH, and its inhibition by Mizoribine confers neuroprotective effects.

6. HSP60 mediates microglial pyroptosis via the TLR4/MyD88/NF-κB signaling axis

**Fig. 9:**
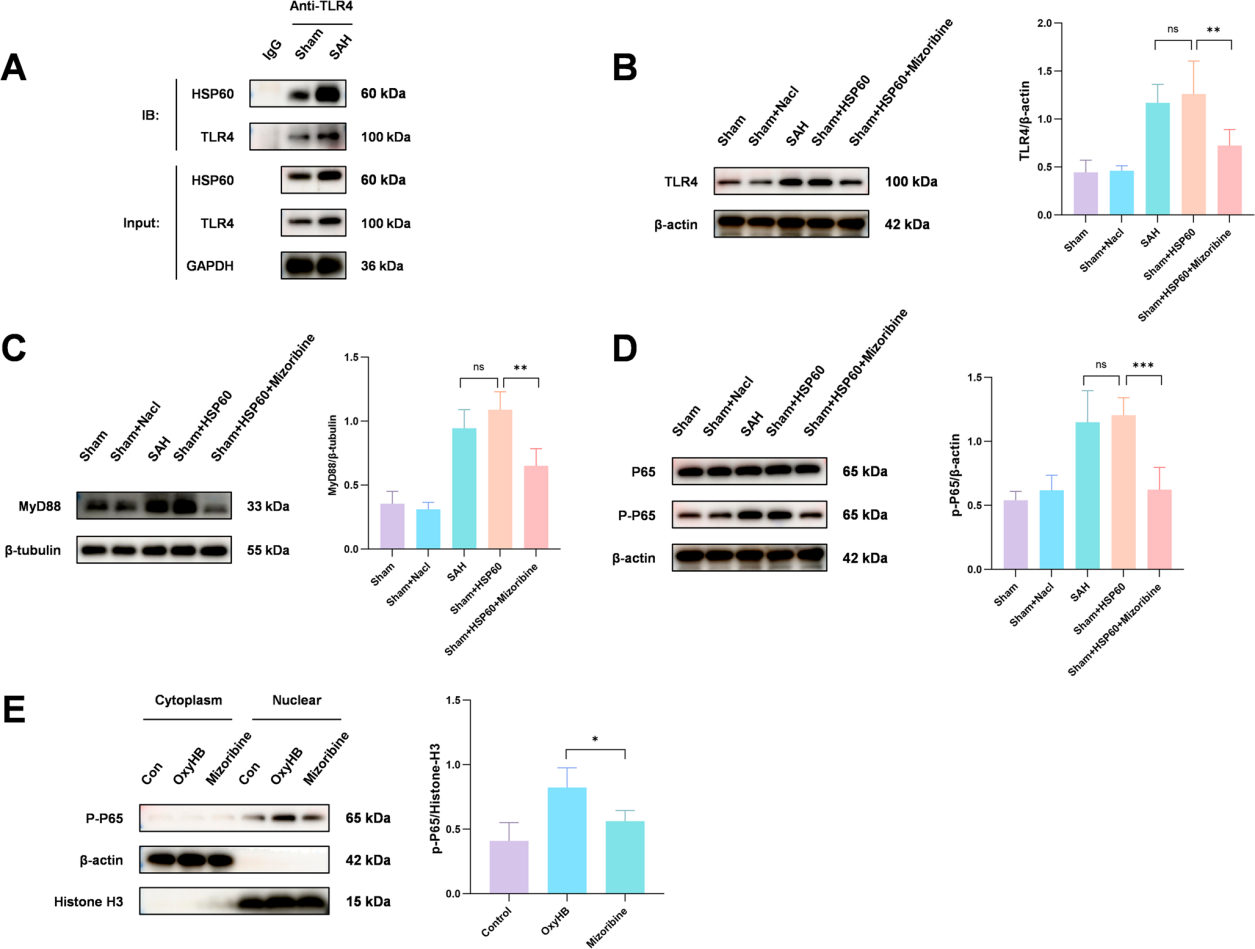
To elucidate the central role of HSP60 in neuroinflammation after SAH, we first confirmed a direct protein-protein interaction between HSP60 and TLR4 by co-immunoprecipitation (Fig. 9A). Building on this interaction, we further validated in vivo through intrathecal injection of recombinant HSP60 protein that it can induce neuroinflammation by activating the TLR4 and its downstream MyD88/NF-κB signaling pathway (Fig. 9B, C, D). To evaluate the therapeutic potential of targeting this pathway, nuclear-cytoplasmic fractionation was performed. The results showed that the HSP60 inhibitor Mizoribine effectively reduced the nuclear accumulation level of phosphorylated NF-κB (p-p65) after SAH (Fig. 9E), mechanistically confirming that HSP60 inhibition blocks the activation of the downstream NF-κB signaling pathway. One-way analysis of variance (ANOVA) followed by Tukey’s post hoc test was performed for comparisons among multiple groups. Data are presented as mean ± SD; ns indicates not significant, *P < 0.05, **P < 0.01, ***P < 0.001

To elucidate the molecular basis of HSP60-mediated TLR4 activation, we first confirmed a direct protein-protein interaction between HSP60 and TLR4 by co-immunoprecipitation (Fig. 9A). Building on this, we successfully established a neuroinflammatory model via intrathecal injection of recombinant HSP60 protein. This treatment specifically activated the TLR4/MyD88/NF-κB pathway, leading to a significant increase in the levels of TLR4, MyD88, and phosphorylated NF-κB (p-p65) in the mouse brain, a phenotype resembling that of the SAH group. Co-administration of the HSP60 inhibitor Mizoribine effectively reversed the activation of this pathway (Fig. 9B). Furthermore, nuclear-cytoplasmic fractionation assays at the cellular level demonstrated that Mizoribine treatment markedly reduced the nuclear level of p-p65 (Fig. 9E), confirming at the cellular level that HSP60 inhibition blocks NF-κB nuclear translocation and transcriptional activation.

**Fig. 10:**
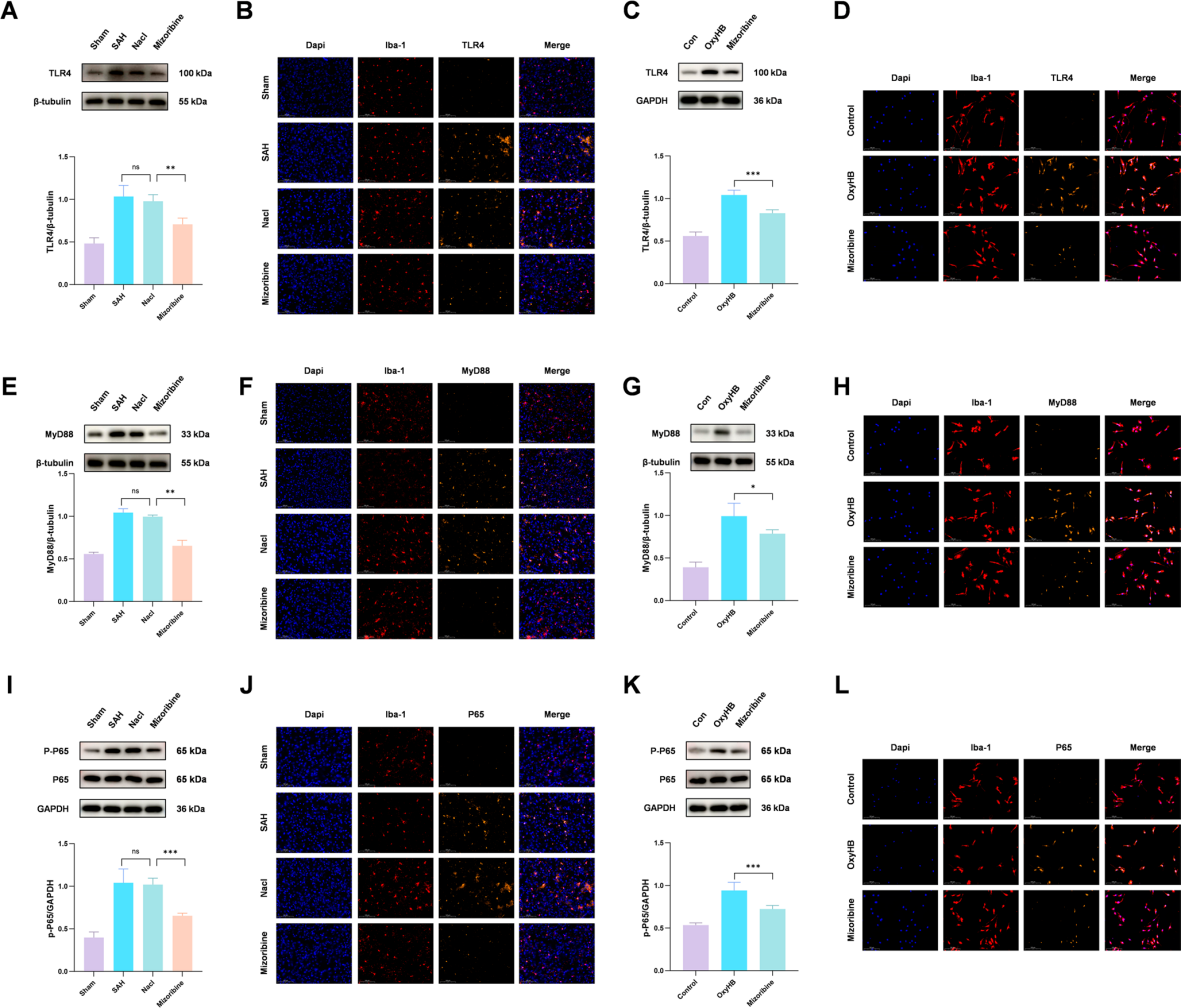
Western blot analysis of the expression levels of key pathway proteins, including TLR4, MyD88, and p-NF-κB, in SAH mice showed a significant increase after SAH, which was markedly reversed by Mizoribine-mediated inhibition of HSP60 (Fig. 10A, E, I). Consistent results were obtained in experiments stimulating microglia with OxyHB (Fig. 10C, G, K). Further co-localization analysis of microglia and the respective pathway proteins by immunofluorescence (Fig. 10B, F, J) demonstrated that Mizoribine treatment significantly attenuated SAH-induced activation of TLR4, MyD88, and p-NF-κB in microglia. Identical results were observed in in vitro experiments (Fig. 10D, H, L). These findings collectively indicate that HSP60 likely participates in the process of microglial pyroptosis after SAH by regulating the TLR4/MyD88/NF-κB signaling pathway. One-way analysis of variance (ANOVA) followed by Tukey’s post hoc test was performed for comparisons among multiple groups. Data are presented as mean ± SD; ns indicates not significant, *P < 0.05, **P < 0.01, ***P < 0.001

Western blot analysis revealed that SAH increased the expression of TLR4, MyD88, and phospho-NF-κB, which was suppressed by Mizoribine (Fig. 10A, E, I). Immunofluorescence co-localization confirmed enhanced expression of these signaling molecules in microglia after SAH, which was attenuated by Mizoribine (Fig. 10B, F, J). In vitro, OxyHB stimulation similarly upregulated TLR4, MyD88, and phospho-NF-κB, and Mizoribine inhibited their expression (Fig. 10C, G, K). Immunofluorescence again supported these findings (Fig. 10D, H, L).

Taken together, our in vivo and in vitro data consistently demonstrate that the upregulation of HSP60 following SAH mediated microglial pyroptosis by activating the TLR4/MyD88/NF-κB signaling pathway, thereby highlighting the therapeutic potential of its inhibition by Mizoribine for alleviating early brain injury.

**Fig. 11:**
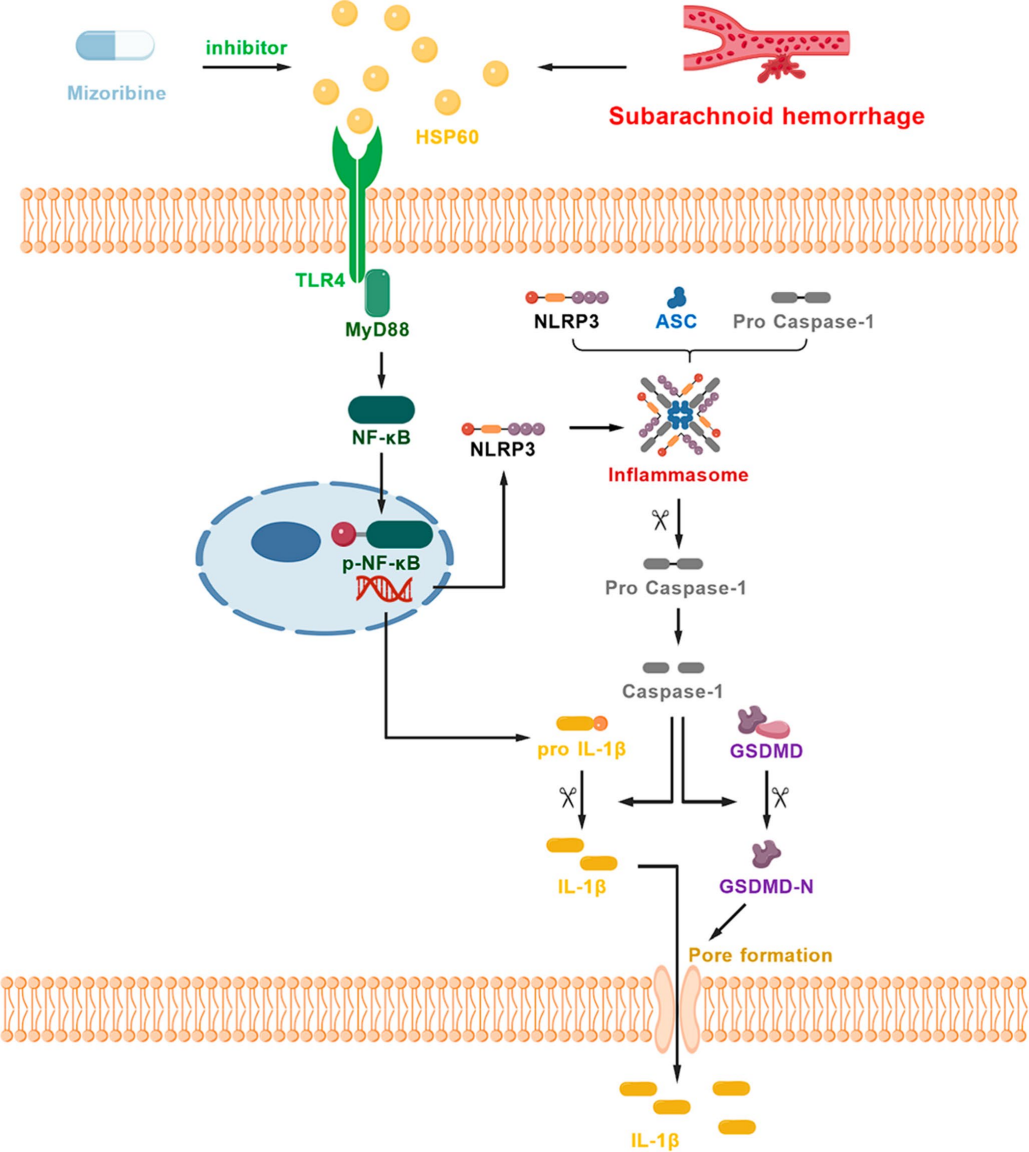
During early brain injury after subarachnoid hemorrhage (SAH), HSP60 expression is significantly upregulated. HSP60 activates the TLR4/MyD88/NF-κB signaling pathway in microglia, inducing upregulation of NLRP3 expression and accumulation of inflammatory cytokine precursors such as pro-IL-1β and pro-IL-18. Elevated NLRP3 further promotes the assembly of the NLRP3 inflammasome, which activates Caspase-1 by cleaving its precursor pro-Caspase-1 into the enzymatically active form Cleaved Caspase-1. Activated Caspase-1 cleaves these cytokine precursors into their mature and biologically active forms, IL-1β and IL-18, and also cleaves gasdermin D (GSDMD), generating its N-terminal fragment (GSDMD-N). GSDMD-N forms pores in the cell membrane, mediating the massive release of mature inflammatory cytokines, ultimately triggering and exacerbating microglial pyroptosis, thereby significantly aggravating the process of early brain injury after SAH. Inhibiting HSP60 with Mizoribine can significantly alleviate HSP60-induced early brain injury after SAH

## Discussion

Subarachnoid hemorrhage (SAH) is a cerebrovascular emergency characterized by high mortality and disability rates [34]. Heat shock protein 60 (HSP60) is an important molecular chaperone primarily localized in mitochondria [35], whose expression is up-regulated under stress conditions. Recent studies indicate that extracellularly released HSP60 can trigger neuroinflammation and lead to neuronal death [36]. Clinical research has also confirmed the involvement of HSP60 in pathological processes such as Alzheimer’s disease (AD) [37, 38]. However, the specific molecular mechanism by which HSP60 regulates microglial activation after SAH remains unclear.

This study aimed to elucidate the role of HSP60 in the early brain injury (EBI) phase following SAH. Given that complete genetic knockout of HSP60 may cause severe developmental defects, the small molecule immunomodulator Mizoribine was selected to selectively inhibit its activity. Experimental results showed that HSP60 expression peaked at 24 h after SAH, and the optimal doses of Mizoribine were determined to be 100 mg/kg in vivo and 80 µM in vitro.

The findings clarify that after SAH, HSP60 binds to Toll-like receptor 4 (TLR4) on the surface of microglia, activating the downstream NF-κB signaling pathway, significantly upregulating NLRP3 inflammasome expression, thereby inducing caspase-1-dependent pyroptosis and promoting the release of pro-inflammatory factors such as IL-1β and IL-18, ultimately exacerbating EBI. Specific inhibition of HSP60 using Mizoribine significantly alleviated neurological deficits, brain edema, blood-brain barrier disruption, and neuronal damage after SAH.

During the EBI process after SAH, microglia, as the primary immune surveillance cells, are rapidly activated. Immunofluorescence staining showed that the density of cells positive for the pro-inflammatory microglial marker CD86[39] was significantly increased in the SAH group, while the proportion decreased markedly after Mizoribine intervention.

As an important endogenous damage-associated molecular pattern (DAMP) [40], HSP60 can be released extracellularly under cellular stress [41], triggering innate immune responses by activating pattern recognition receptors such as the TLR4/CD14 complex [42]. TLR4 activation can induce nuclear factor κB (NF-κB) activation, promoting the transcription of the NLRP3 inflammasome and pro-IL-1β[43–45]. This study demonstrates that upregulation of HSP60 expression after SAH activates the TLR4/MyD88/NF-κB signaling axis, whereas inhibition of HSP60 with Mizoribine led to a dose-dependent decrease in the expression of key molecules in this signaling axis.

Pyroptosis is a form of programmed cell death mediated by Gasdermin proteins, characterized by cell membrane perforation and inflammatory factor release [18, 46]. The NLRP3 inflammasome can activate caspase-1, which then cleaves Gasdermin D (GSDMD) and promotes the maturation of IL-1β and IL-18[48, 49]. This study found that HSP60 promotes NLRP3 inflammasome assembly via the TLR4/MyD88/NF-κB signaling pathway, activates caspase-1, and induces microglial pyroptosis. After Mizoribine intervention, the number of cells positive for pyroptosis-related molecules in microglia decreased, while degradation of tight junction proteins was reduced, blood-brain barrier permeability improved, and ultimately neurological function was ameliorated [52, 53].

It is undeniable that this study still has certain limitations. The work primarily focused on the impact of HSP60 on microglia and has not yet systematically investigated its interactions with other neural cells such as neurons and astrocytes in the context of SAH. Furthermore, although a significant increase in HSP60 expression post-SAH was observed, its precise release concentration and the threshold for triggering neuroinflammation remain to be clarified. Given that our current research is confined to the level of pharmacological inhibition at the protein level, future studies need to further explore the more detailed mechanisms of HSP60 after subarachnoid hemorrhage at the genetic level to uncover the underlying mechanisms. Nevertheless, this study is the first to reveal the crucial role of HSP60 in inducing microglial pyroptosis via the TLR4/MyD88/NF-κB-NLRP3 signaling axis in early brain injury after SAH. It also demonstrates that Mizoribine can effectively mitigate neuroinflammation and brain damage by inhibiting HSP60, thereby providing a novel molecular target and experimental basis for neuroprotective therapy in SAH.

## Conclusions

This study demonstrates that elevated heat shock protein 60 (HSP60) following subarachnoid hemorrhage (SAH) activates the TLR4/MyD88/NF-κB signaling axis, upregulates NLRP3 inflammasome expression, and catalyzes the cleavage of pro-caspase-1 into active caspase-1. Activated caspase-1 promotes the release of mature IL-1β and IL-18 by cleaving their precursor forms (pro-IL-1β and pro-IL-18), while also inducing the oligomerization of the N-terminal domain of Gasdermin D (GSDMD-N), ultimately driving microglial pyroptosis. This process significantly exacerbates neuroinflammatory responses and aggravates brain injury after SAH. Critically, intervention with Mizoribine to inhibit HSP60 effectively blocks this cascade, significantly reduces microglial pyroptosis, and ameliorates neuroinflammation-related pathological damage (Figure 11).

## Supplementary Information

Below is the link to the electronic supplementary material.

Supplementary Material 1 (DOCX 3.16 MB)

Supplementary Material 2 (DOCX 1.03 MB)

## Data Availability

No datasets were generated or analysed during the current study. The original data supporting the findings of this study, including uncropped Western blot images, raw immunofluorescence and electron microscopy images, are available from the corresponding author upon reasonable request.

## Abbreviations

SAH: Subarachnoid Hemorrhage
EBI: Early Brain Injury
DAMPs: Damage-Associated Molecular Patterns
OxyHB: Oxyhemoglobin
HSP60: Heat Shock Protein 60
IL-1β: Interleukin-1β
IL-18: Interleukin-18
TLR4: Toll-Like Receptor 4
MyD88: Myeloid Differentiation primary response 88
NF-κB: Nuclear Factor kappa-light-chain-enhancer of activated B cells
GSDMD: Gasdermin D
Iba1: Ionized Calcium-binding adapter molecule 1
NeuN: Neuronal Nuclei
CD86: Cluster of Differentiation 86
CD206: Cluster of Differentiation 206 ( Mannose Receptor )
IF: Immunofluorescence
IHC: Immunohistochemistry
TEM: Transmission Electron Microscopy
ELISA: Enzyme-Linked Immunosorbent Assay
TUNEL: Terminal deoxynucleotidyl Transferase dUTP Nick-End Labeling
