# Supplementary material for: HSP60 Mediates NLRP3 Inflammasome-Dependent Microglial Pyroptosis Via the TLR4/MyD88/NF-κB Signaling Axis After Subarachnoid Hemorrhage
Source: Inflammation. 2026 Jan 12;49(1):41. doi: 10.1007/s10753-025-02442-x (PMC12862021; doi:10.1007/s10753-025-02442-x)
Supplement: Supplementary file 1 — Supplementary Material 1 (DOCX 3.16 MB) [file 10753_2025_2442_MOESM1_ESM.docx]

**Figure2.A**

**
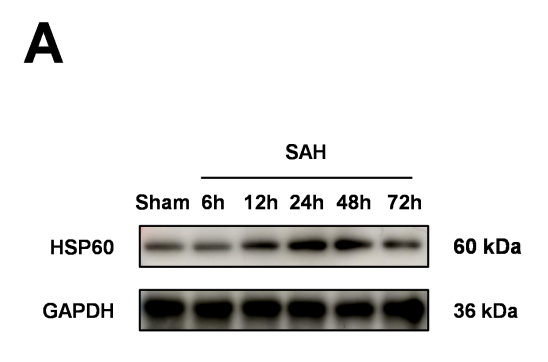
**


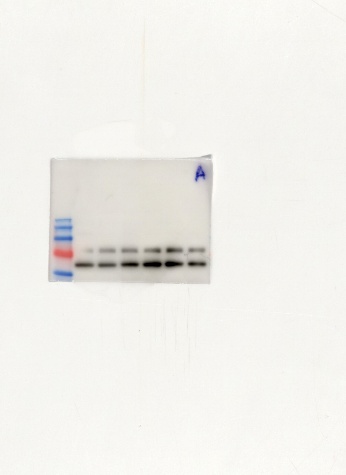

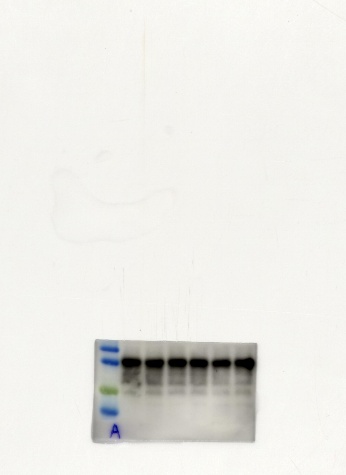

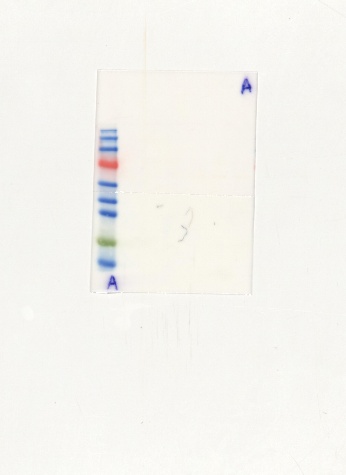


**Figure2.C**


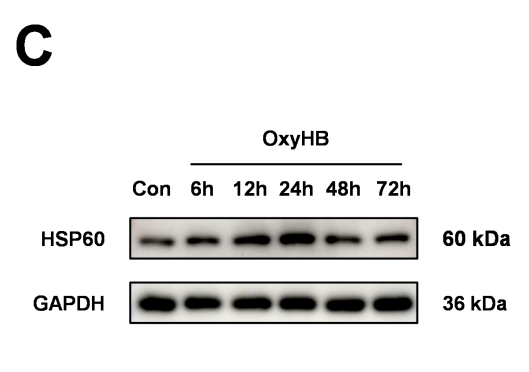


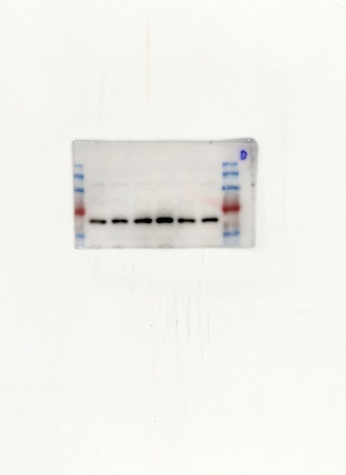

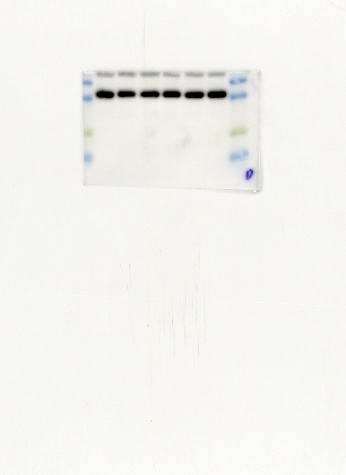

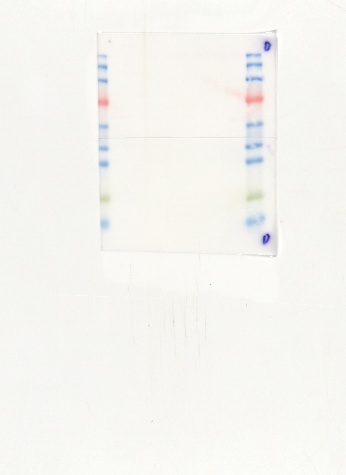


**Figure2.D**

**
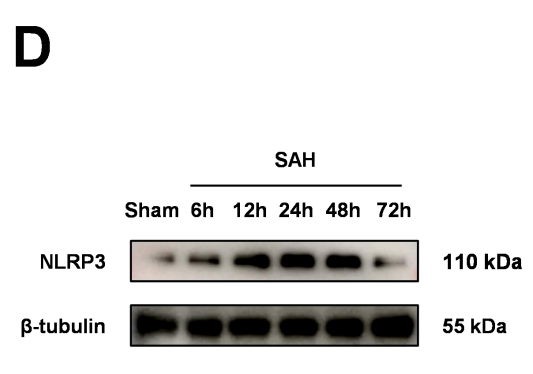
**


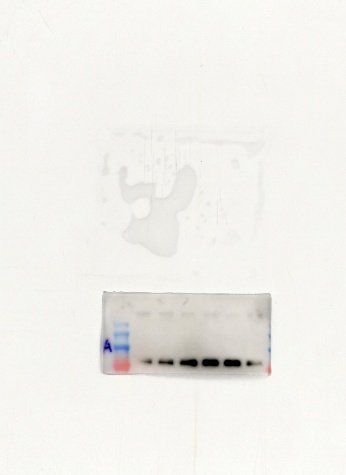

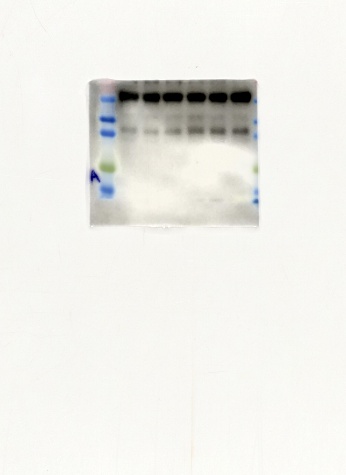

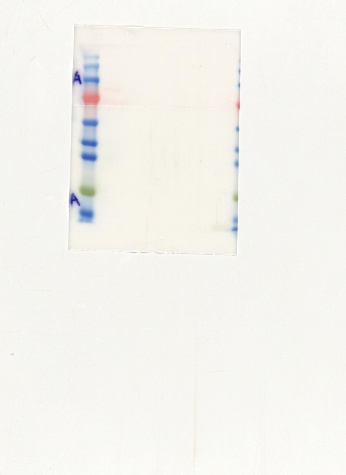


**Figure2.F**

**
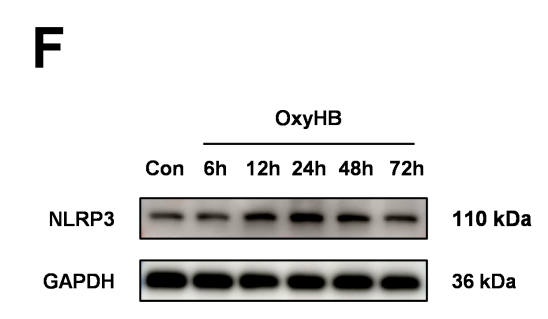
**


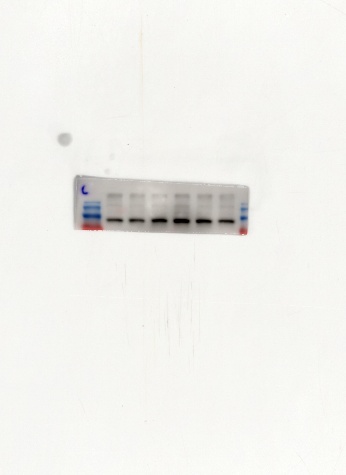

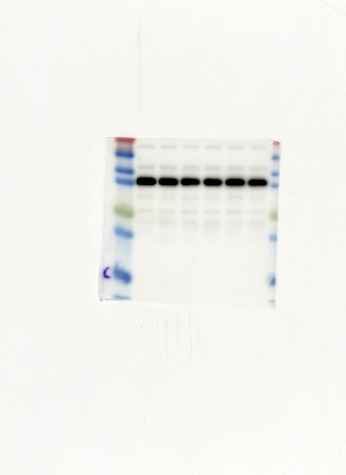

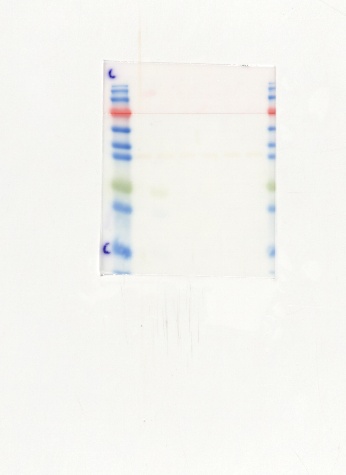


**Figure3.A**

**
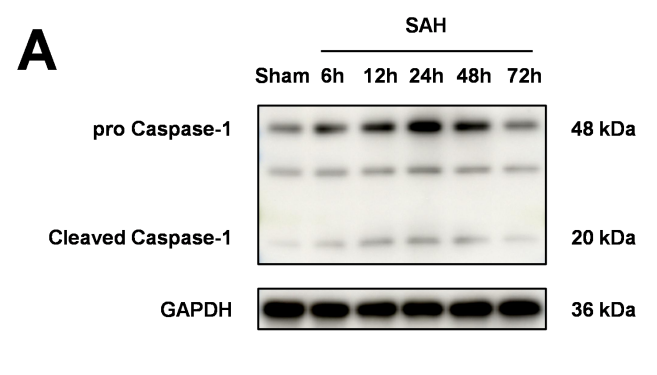
**


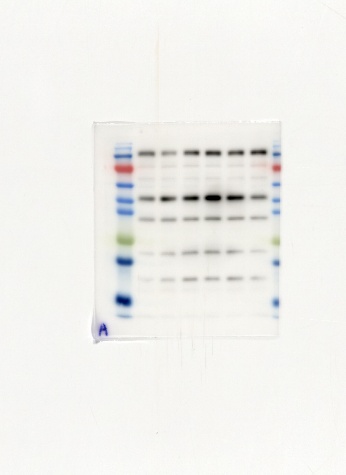

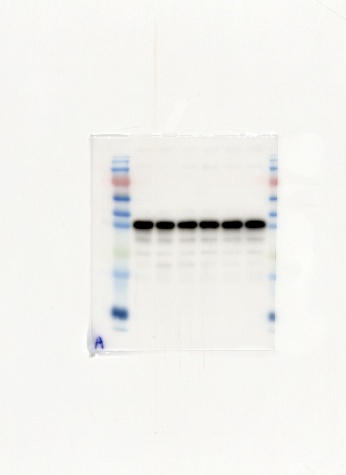

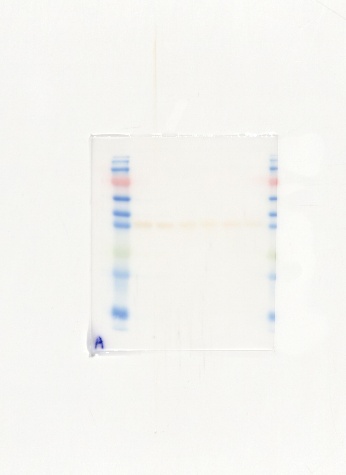


**Figure3.B**

**
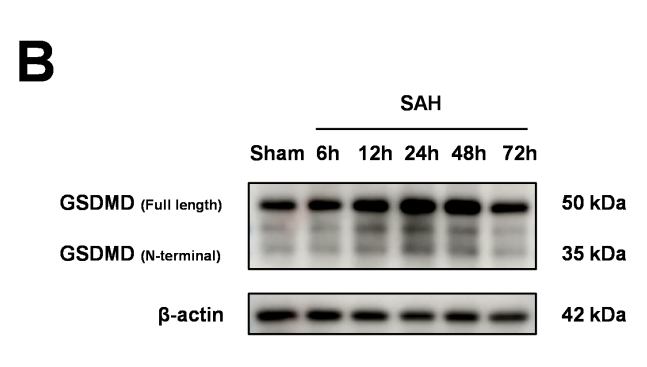
**


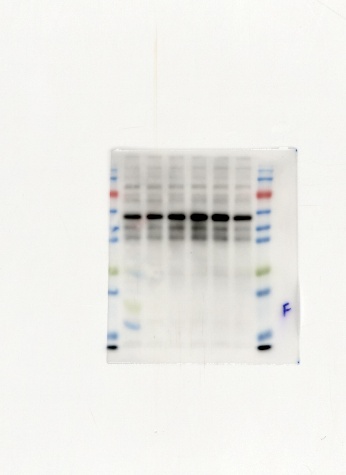

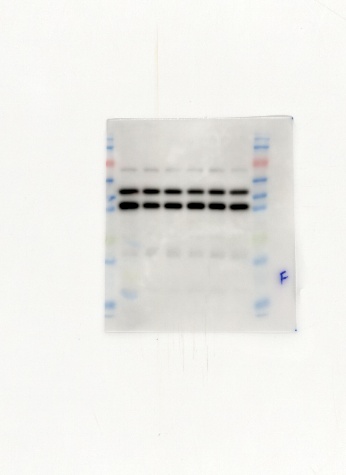

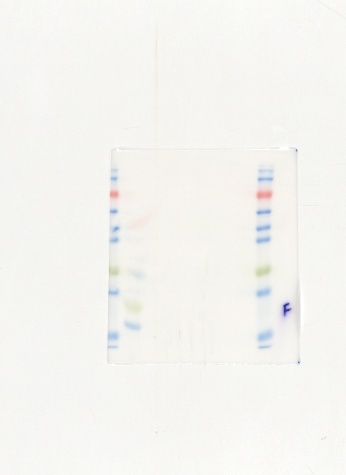


**Figure3.C**

**
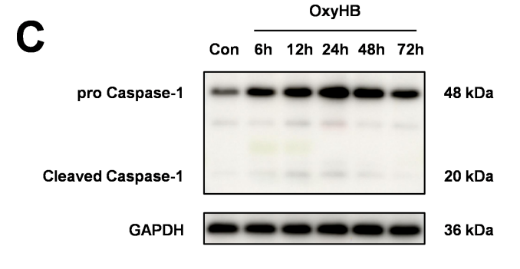
**


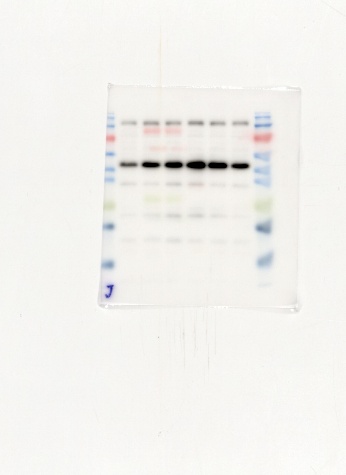

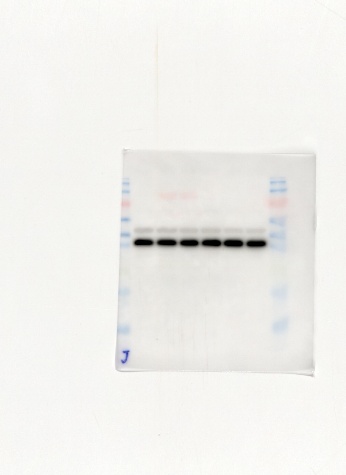

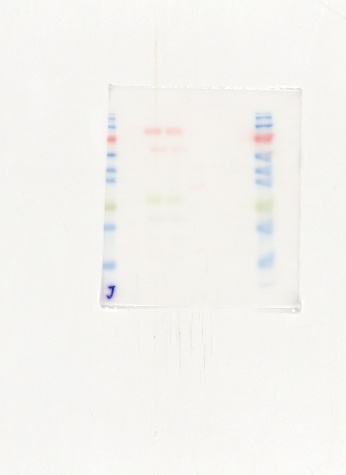


**Figure3.D**

**
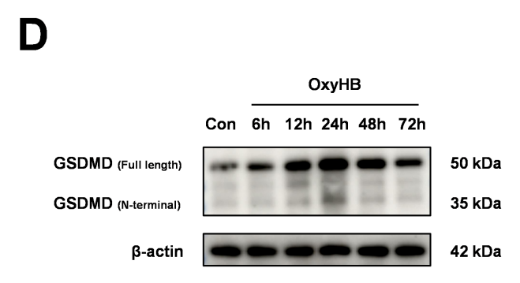
**


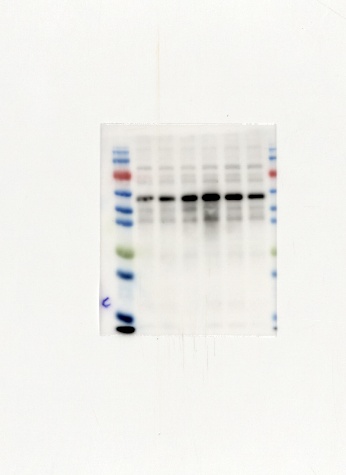

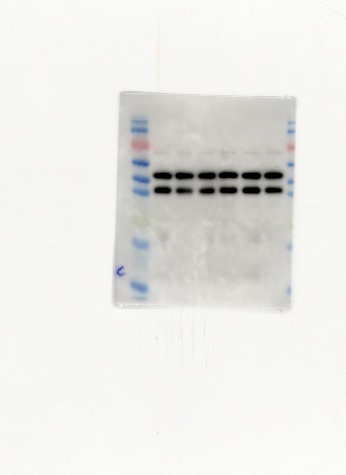

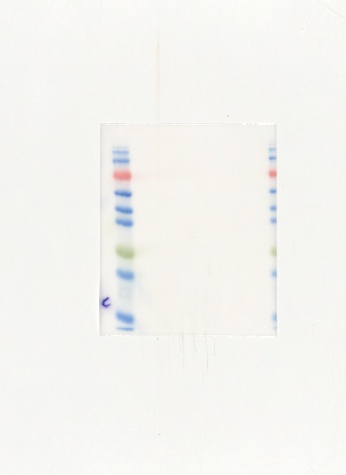


**Figure4.A**

**
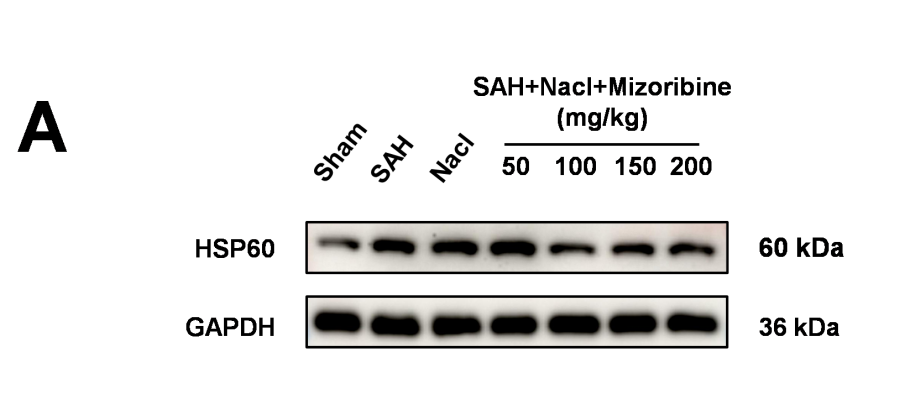
**


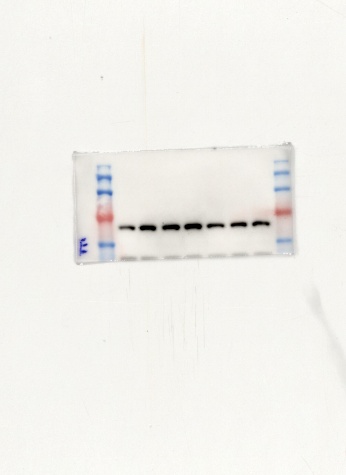

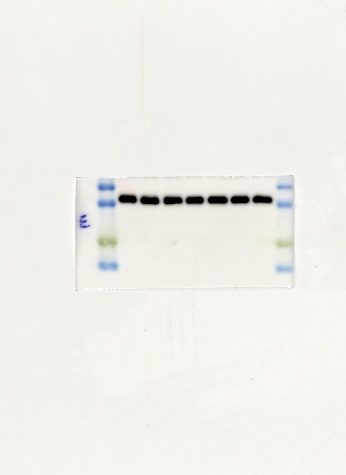

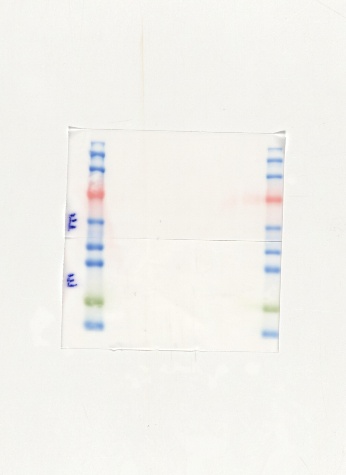


**Figure4.C**

**
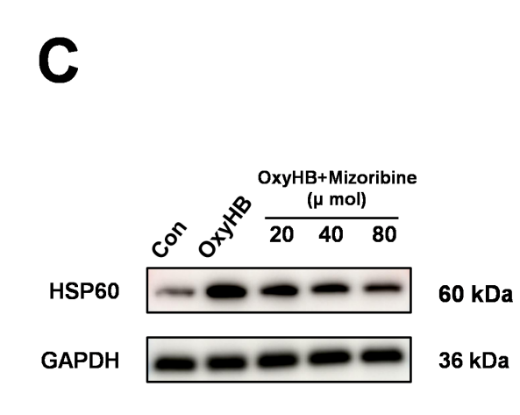
**


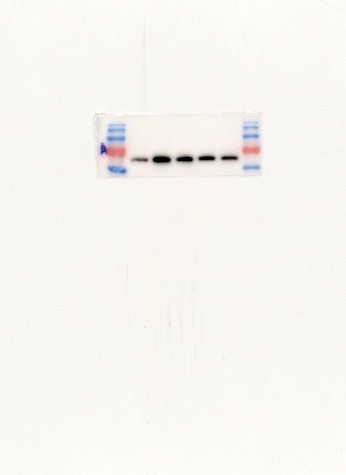

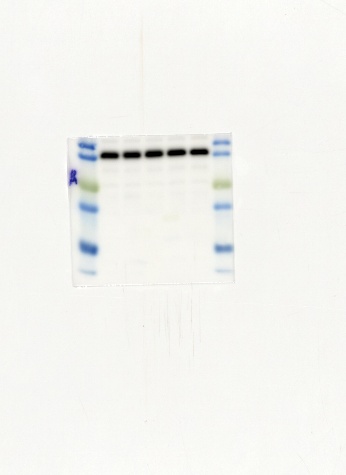

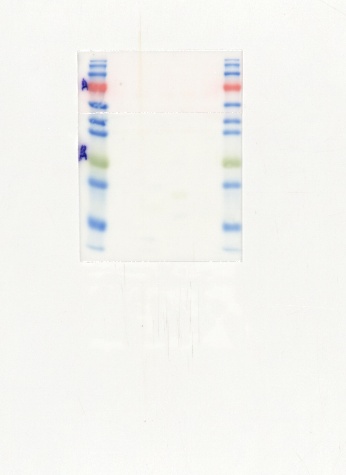


**Figure5.A**

**
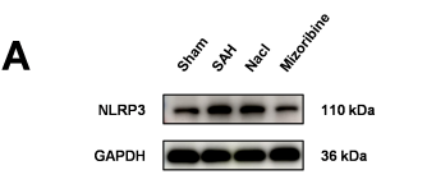
**


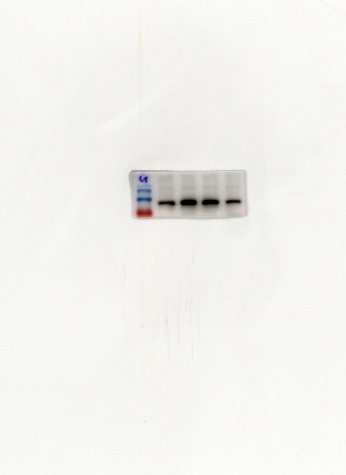

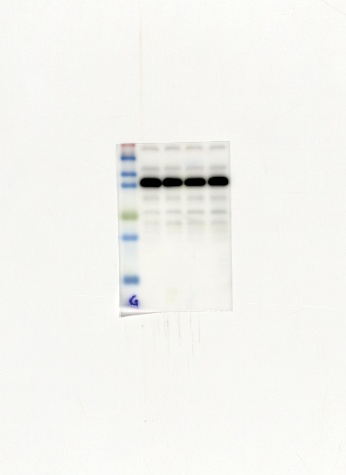

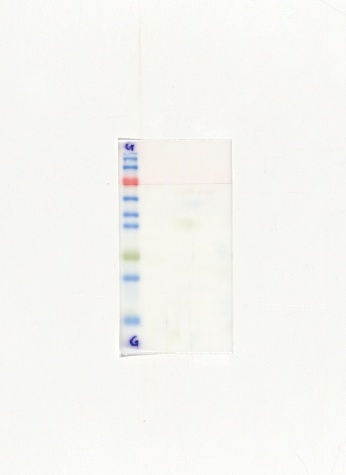


**Figure5.E**

**
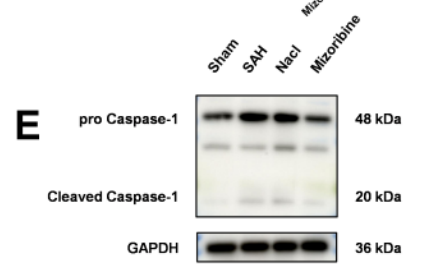
**


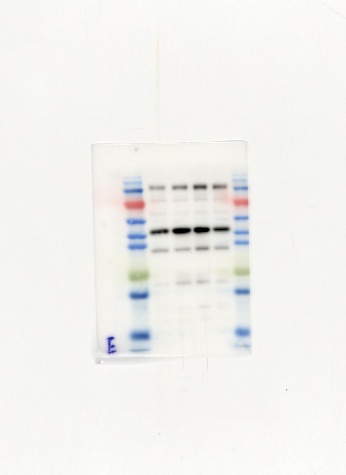

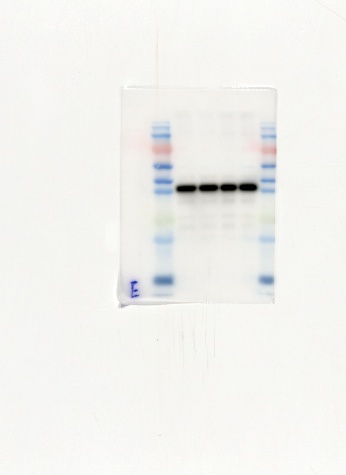

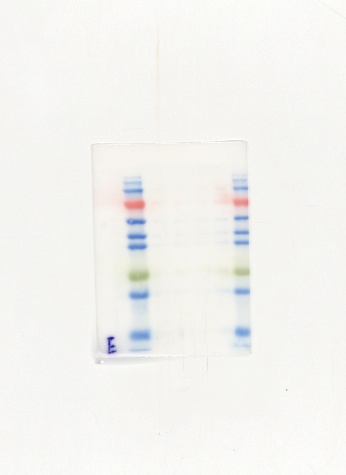


**Figure5.I**

**
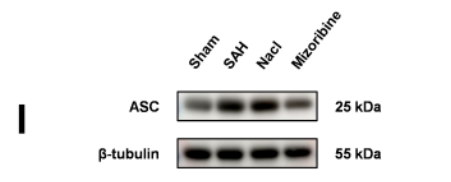
**


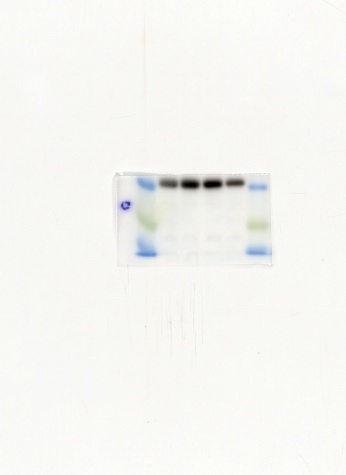

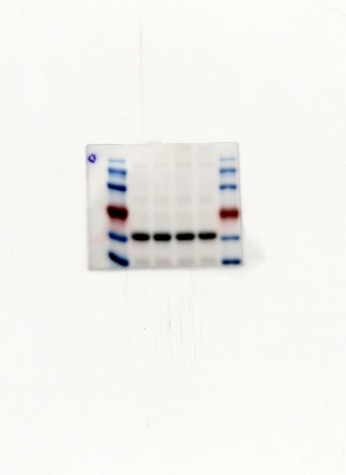

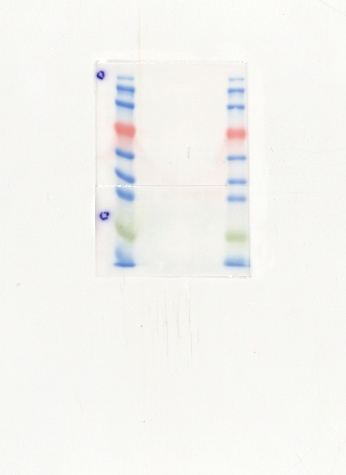


**Figure5.C**

**
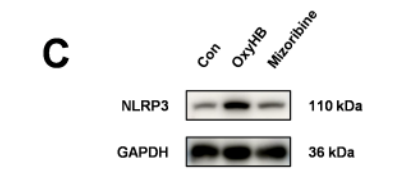
**


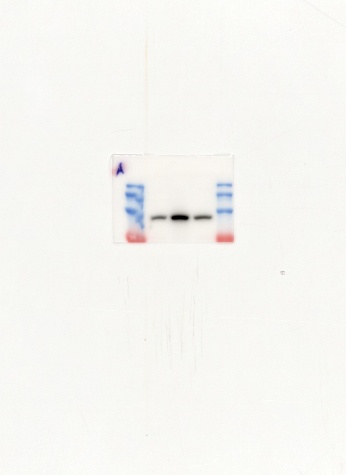

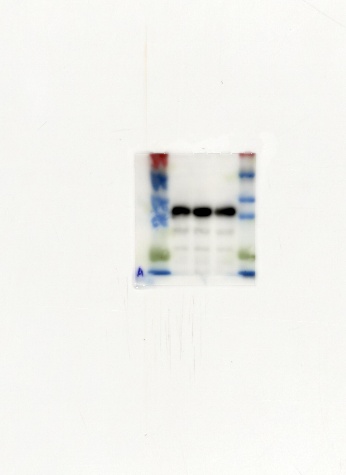

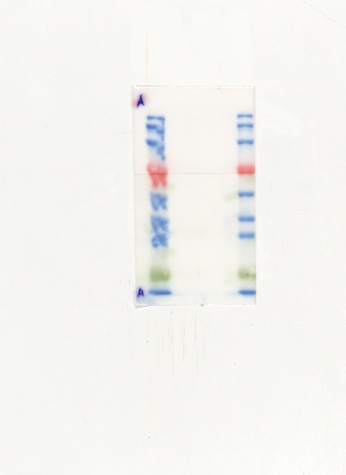


**Figure5.G**

**
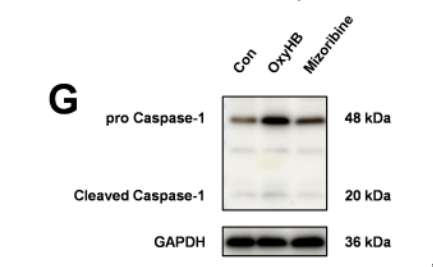
**


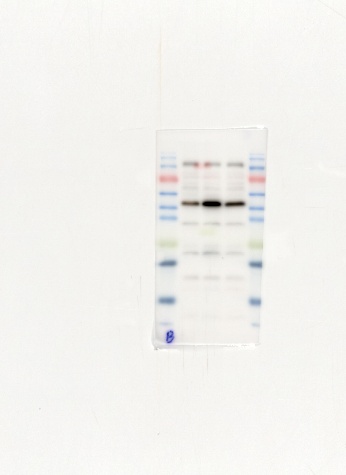

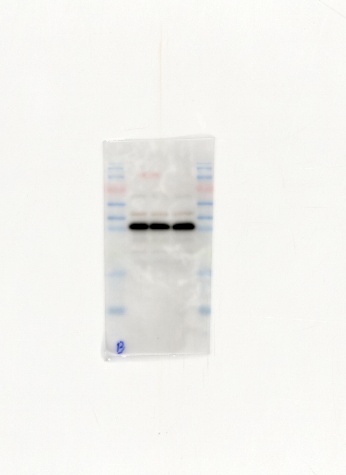

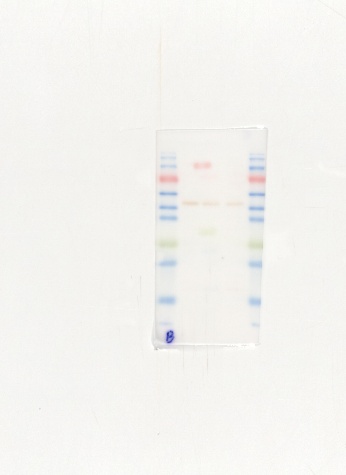


**Figure5.K**

**
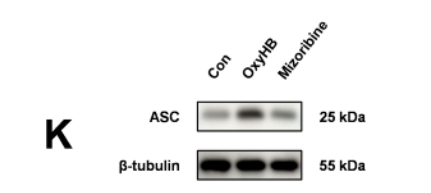
**


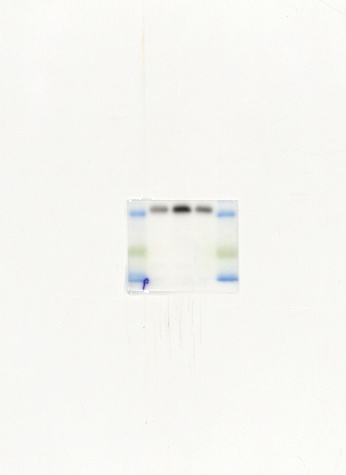

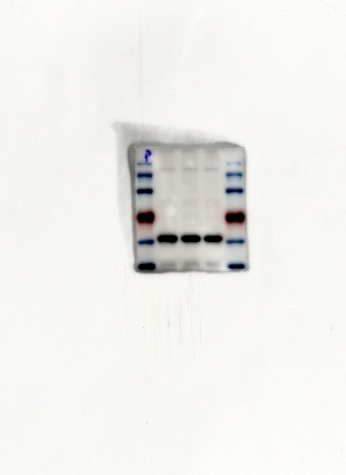

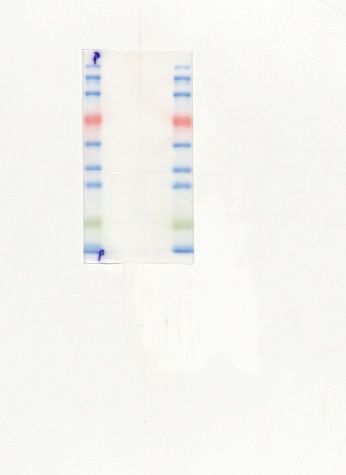


**Figure6.A**

**
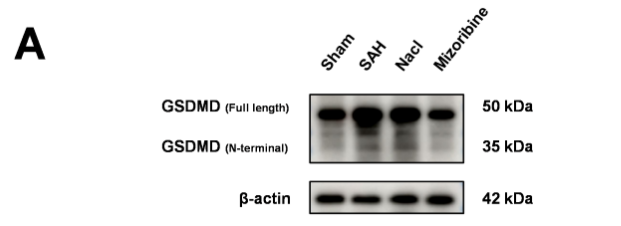
**


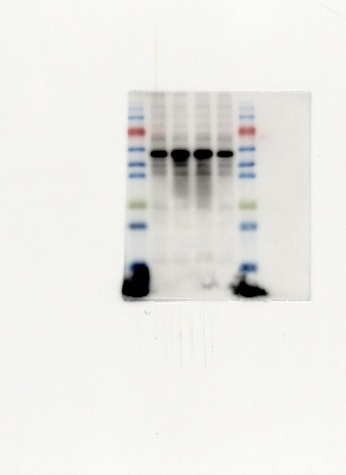

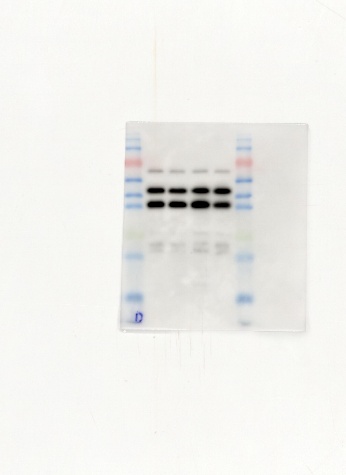

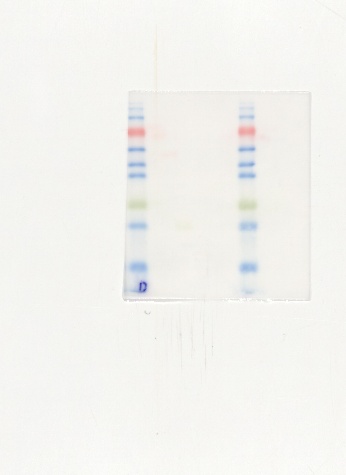


**Figure6.C**

**
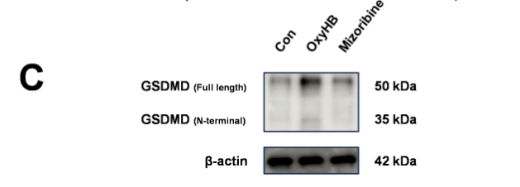
**


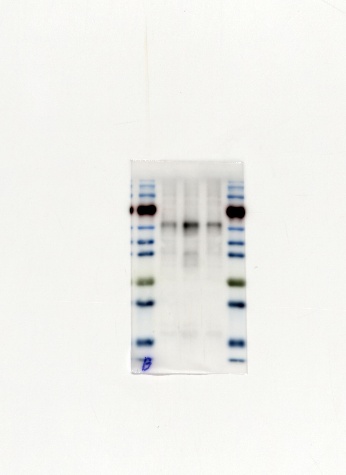

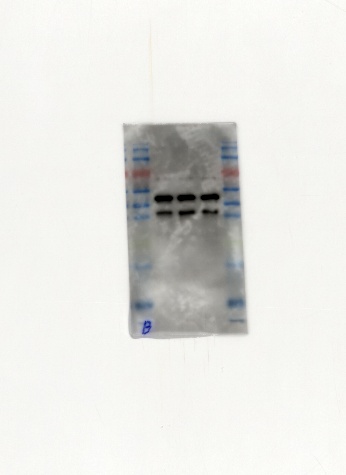

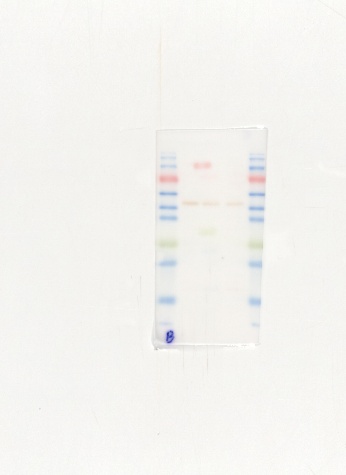


**Figure7.A**

**
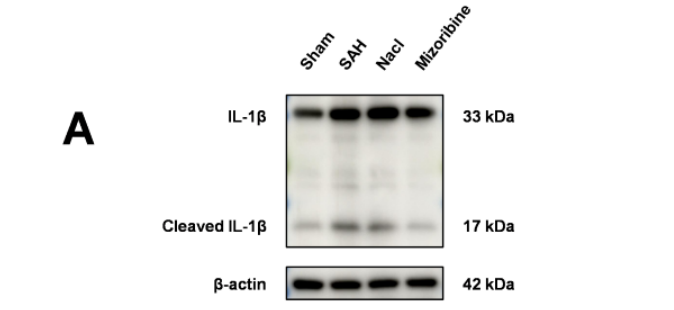
**


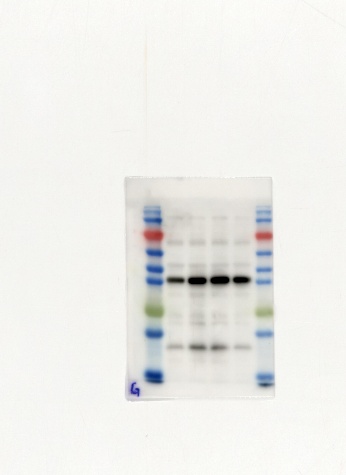

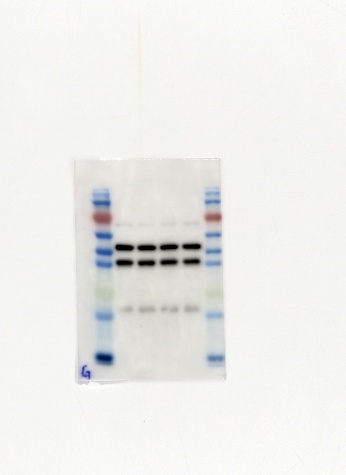

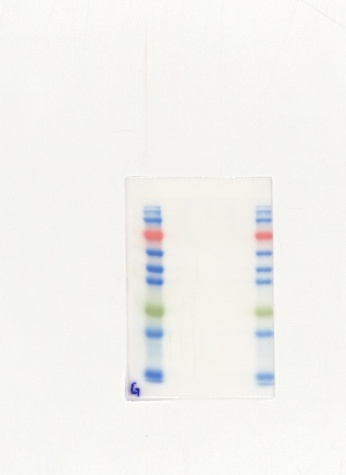


**Figure7.B**

**
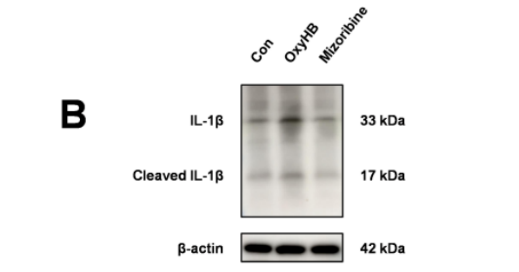
**


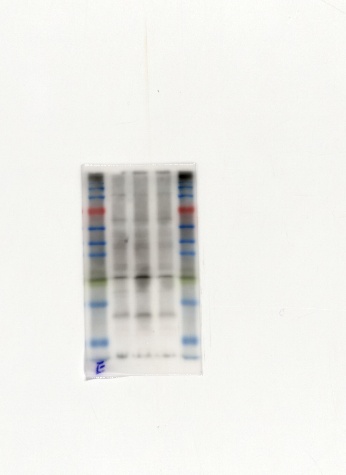

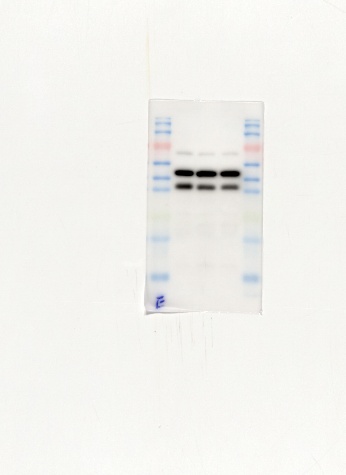

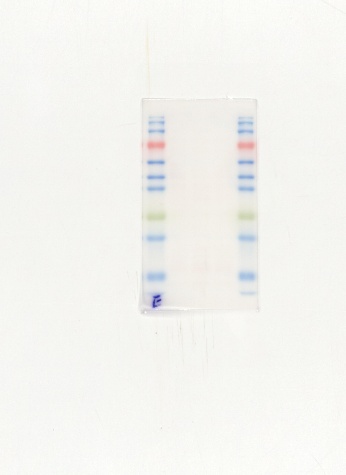


**Figure8.A**

**
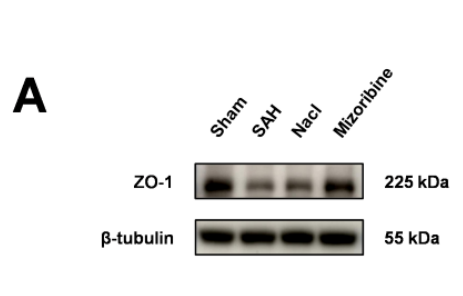
**


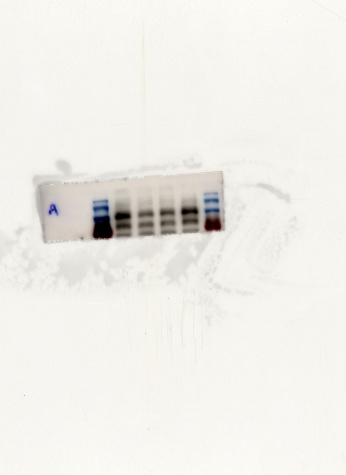

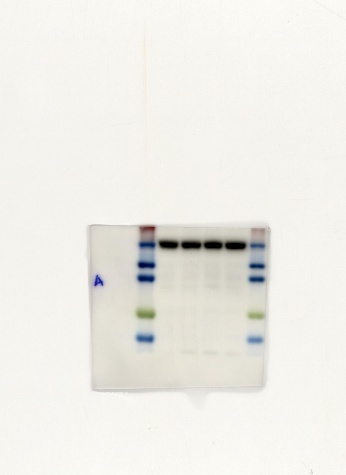

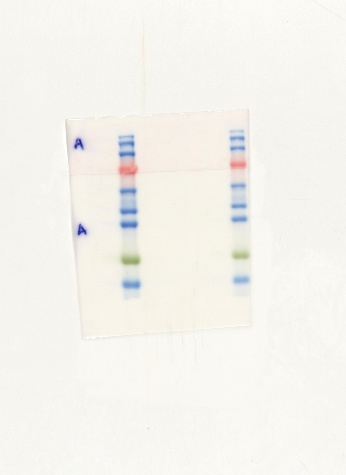


**Figure8.B**

**
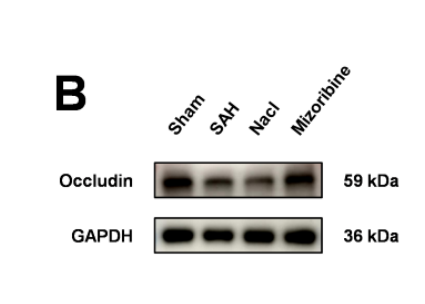
**


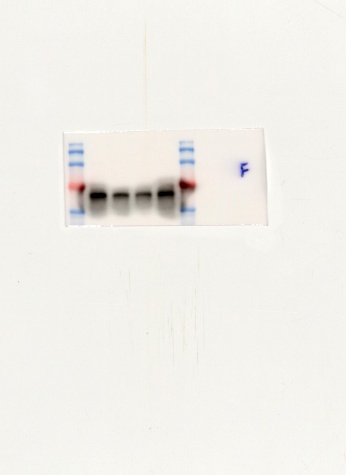

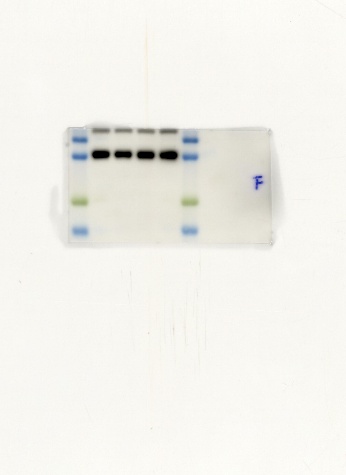

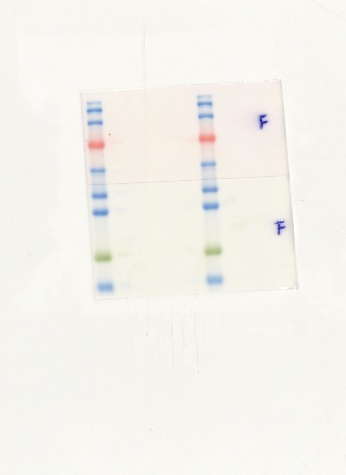


**Figure9.A**

**
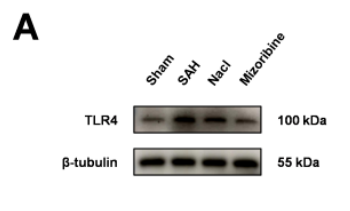
**


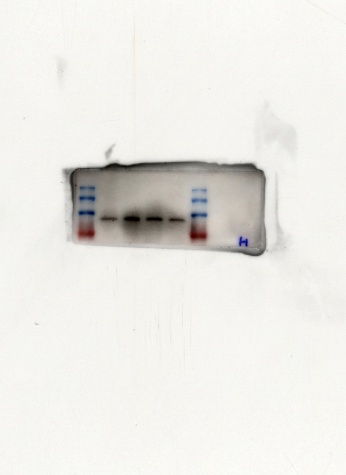

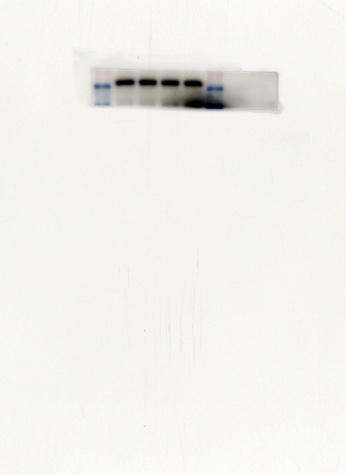

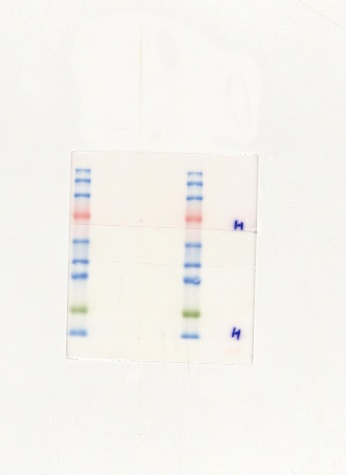


**Figure9.E**

**
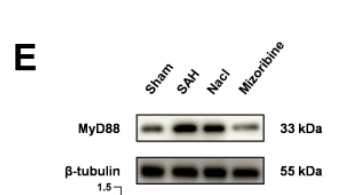
**


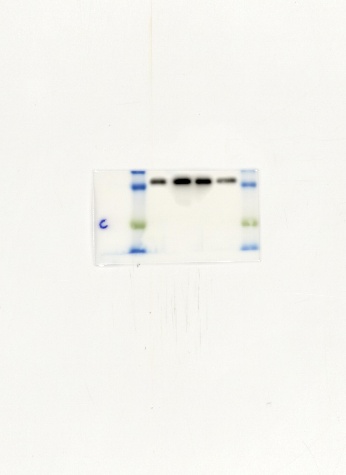

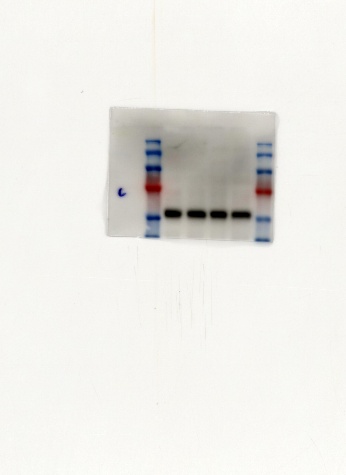

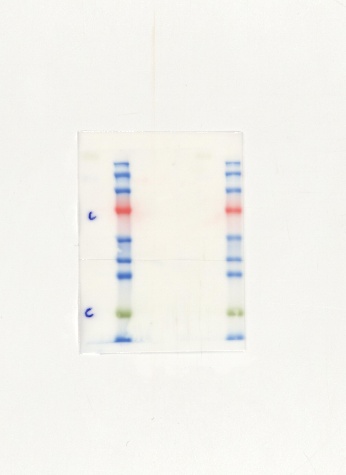


**Figure9.I**

**
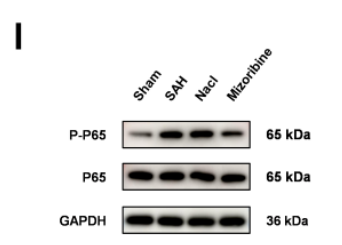
**


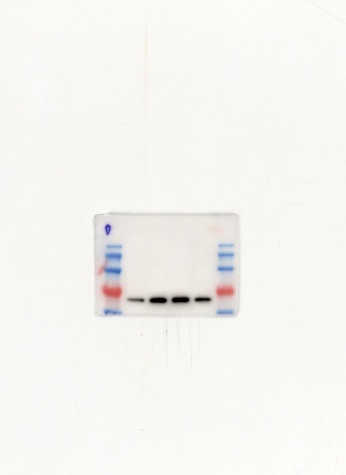

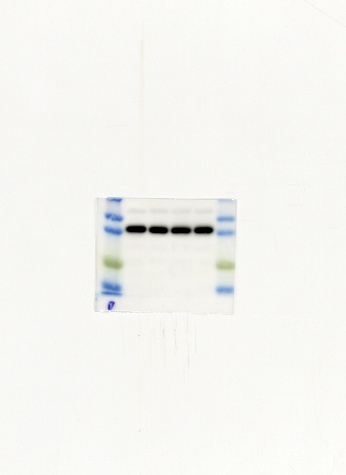

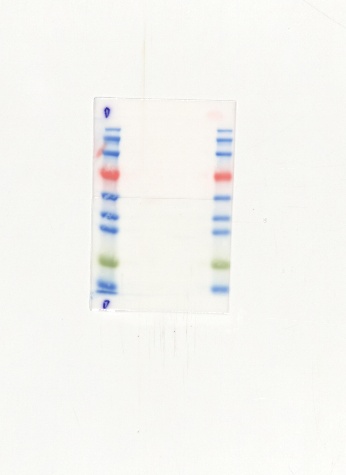


**Figure9.C**

**Figure9.G**

**Figure9.K**
