# Supplementary figures and images for: HSP60 Mediates NLRP3 Inflammasome-Dependent Microglial Pyroptosis Via the TLR4/MyD88/NF-κB Signaling Axis After Subarachnoid Hemorrhage
Source: Inflammation. 2026 Jan 12;49(1):41. doi: 10.1007/s10753-025-02442-x (PMC12862021; doi:10.1007/s10753-025-02442-x)

**figure4E**

**
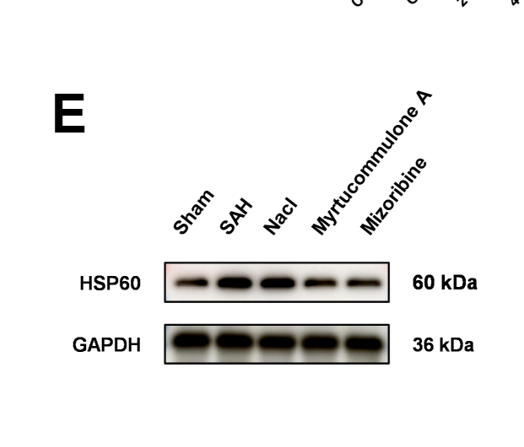
**


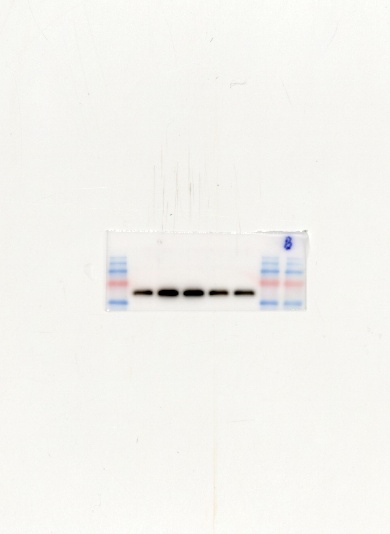

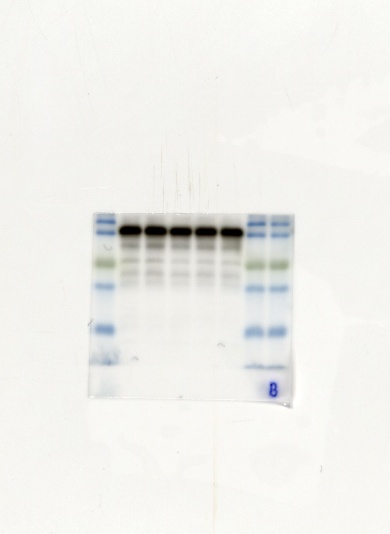

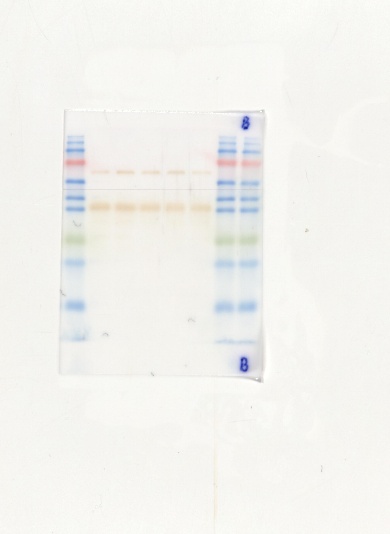


**figure9A**
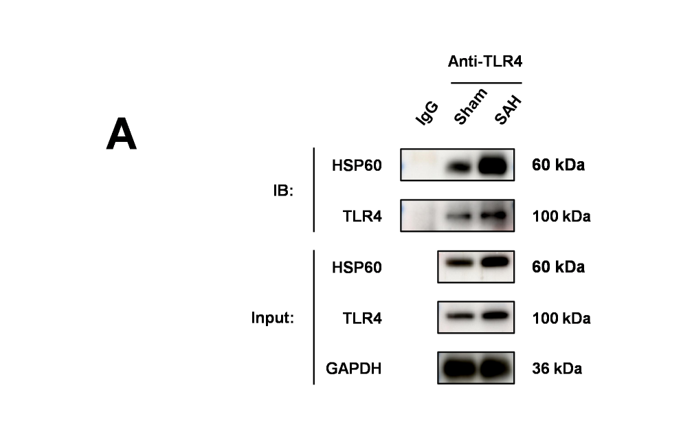


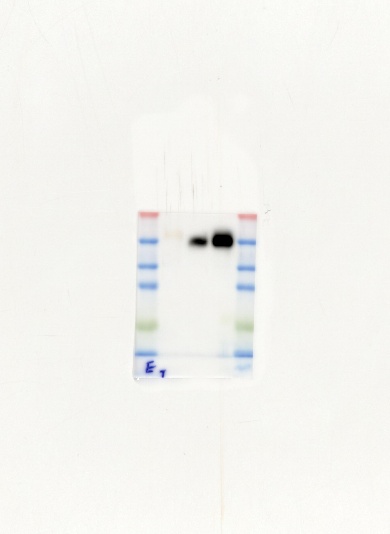

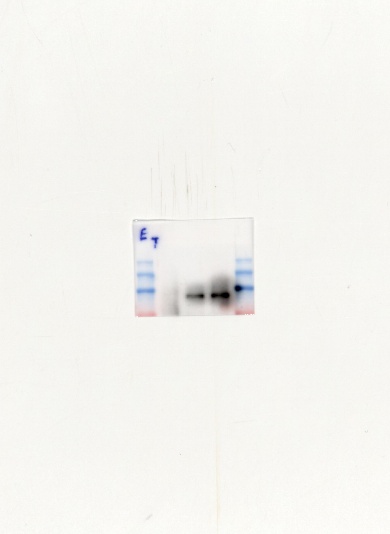

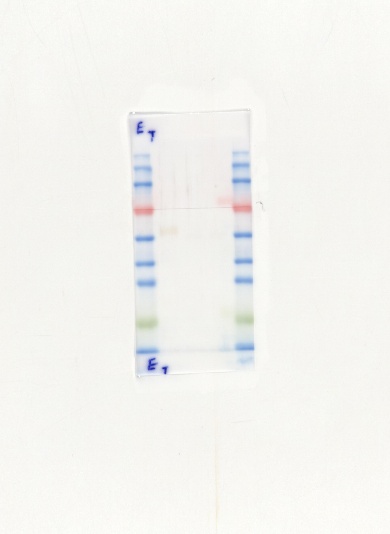


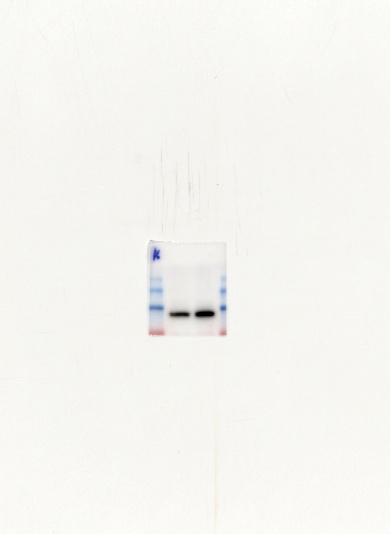

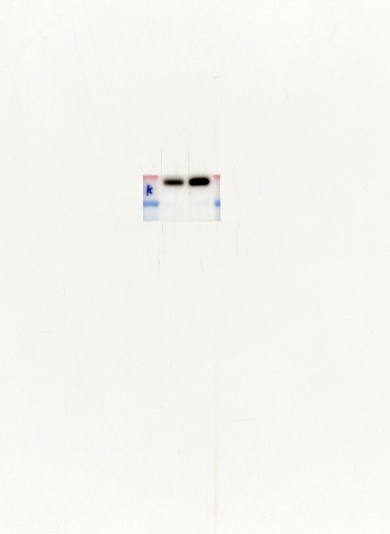

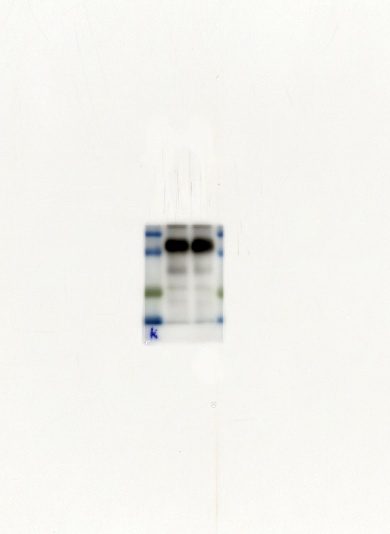


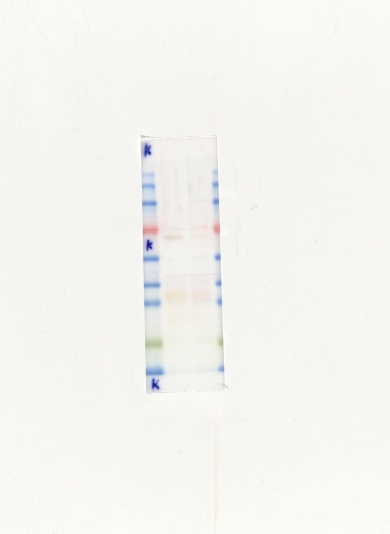


**figure9B**


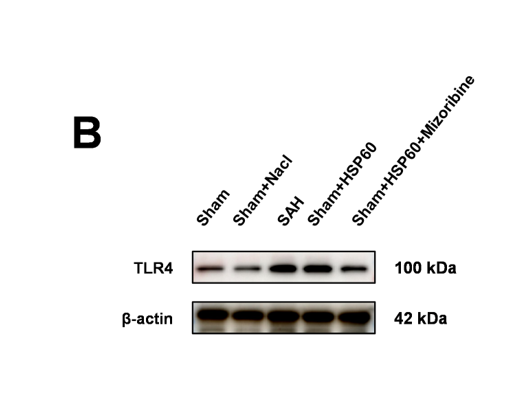


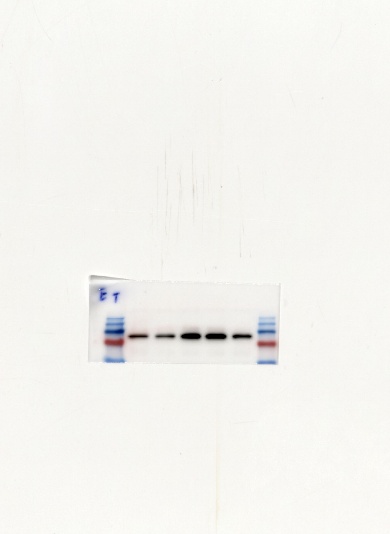

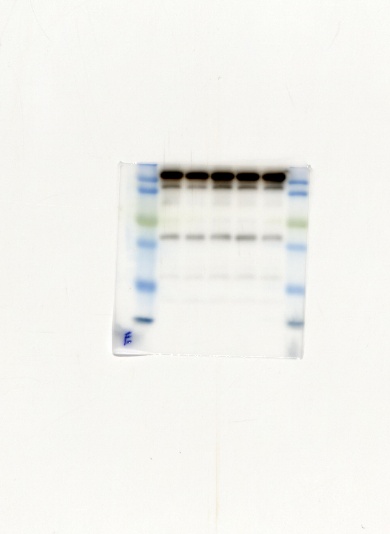

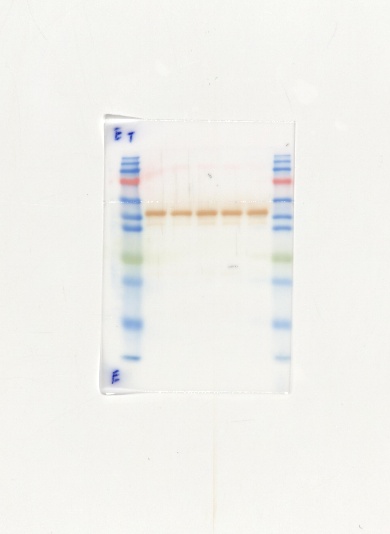


**figure9C**

**
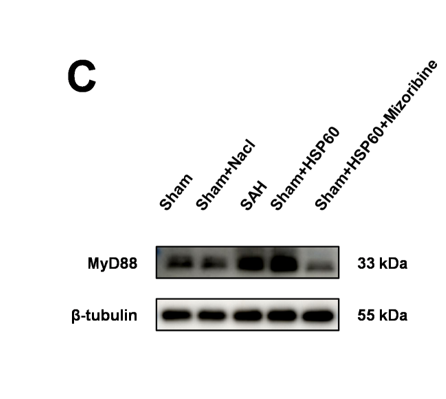
**


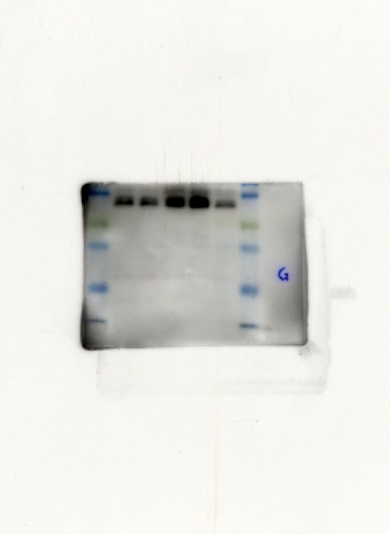

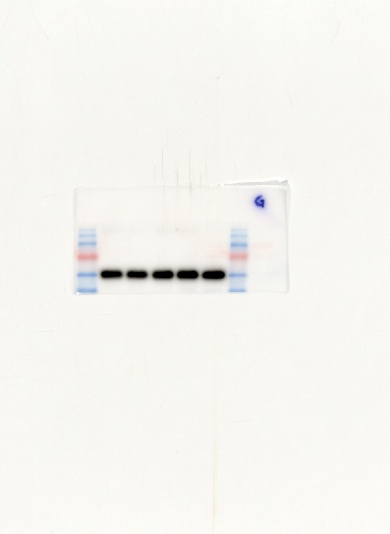

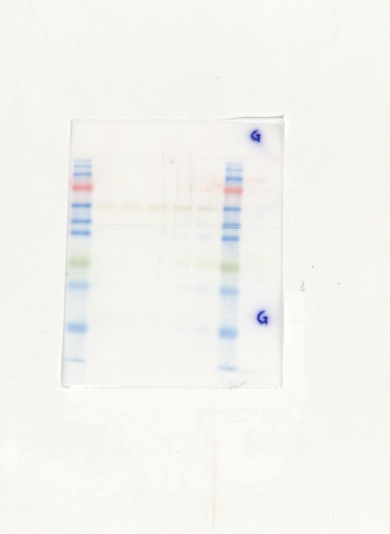


**figure9D**

**
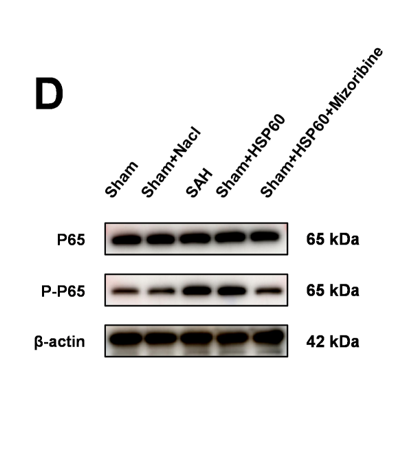
**


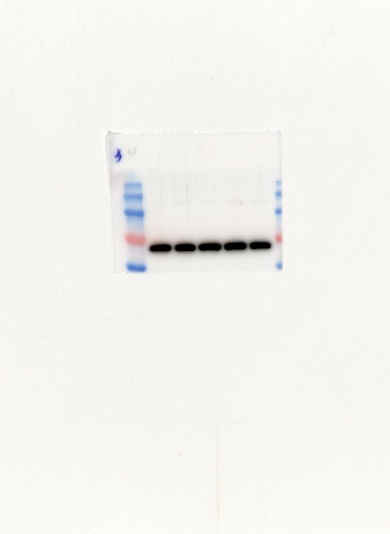

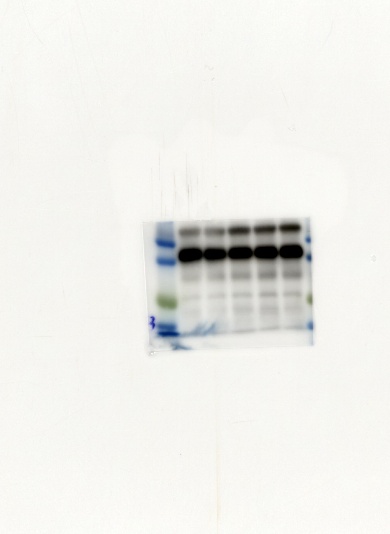

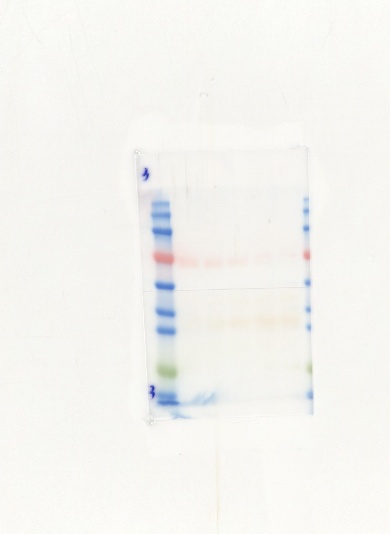

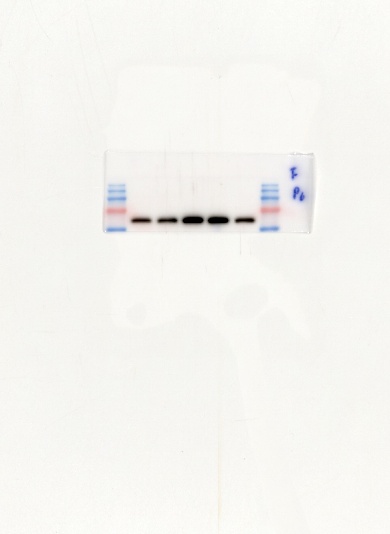

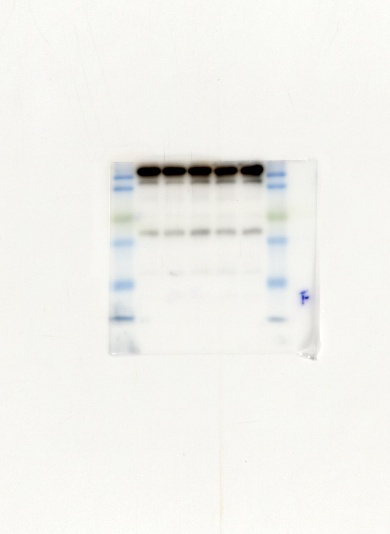

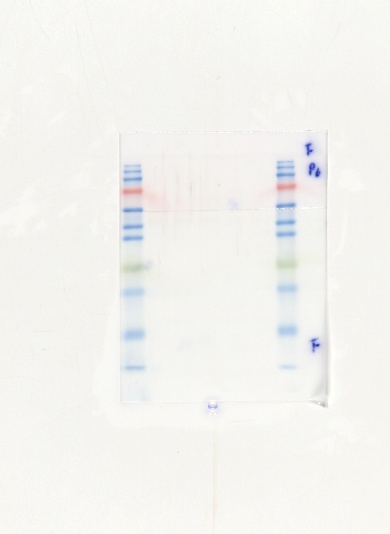


**figure9E**

**
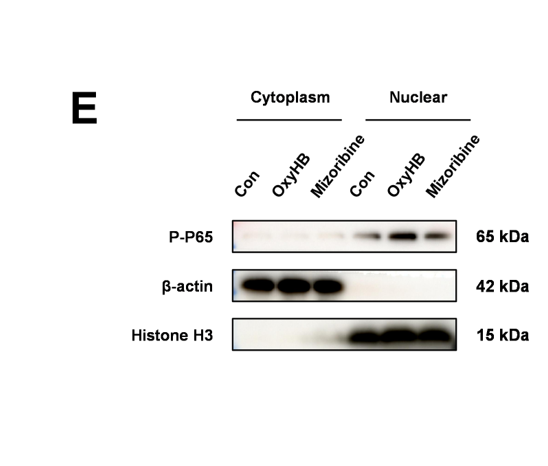
**


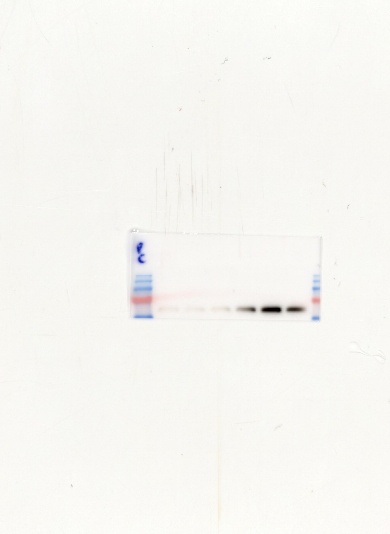

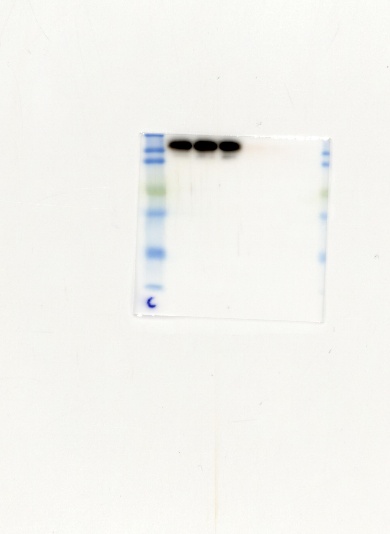

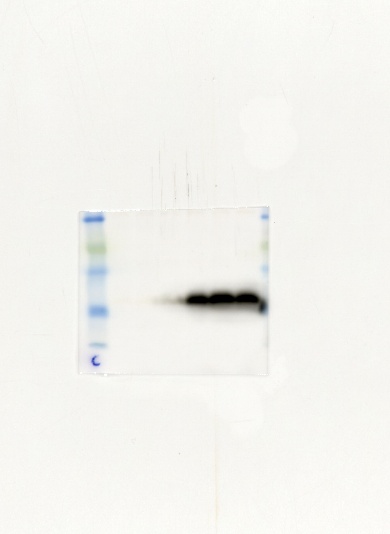

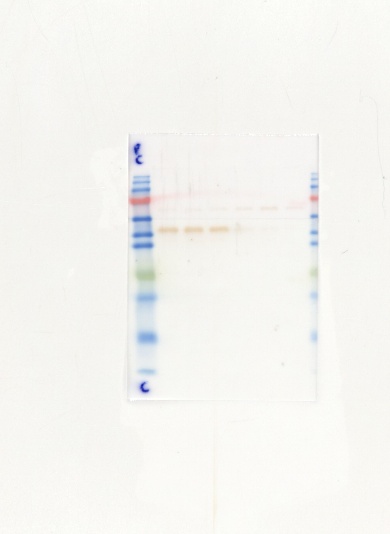

Supplement: Supplementary file 2 — Supplementary Material 2 (DOCX 1.03 MB) [file 10753_2025_2442_MOESM2_ESM.docx]
